# Global Epidemiology of Diabetic Foot: A Systematic Review and Meta‐Analysis

**DOI:** 10.1002/dmrr.70223

**Published:** 2026-08-31

**Authors:** Massimo Carollo, Ylenia Ingrasciotta, Salvatore Crisafulli, Francesco Maccarrone, Daniela Pellegrino, Alberto Francescon, Edoardo Mannucci, Andrea Fontana, Gianluca Trifirò

**Affiliations:** ^1^ Department of Diagnostics and Public Health University of Verona Verona Italy; ^2^ Academic Spin‐off ‘Innovative Solutions for Medical Prediction and Big Data Integration in Real World Setting Srl‐INSPIRE SRL’ University of Messina Messina Italy; ^3^ Department of Biomedical, Experimental and Clinical Sciences University of Florence Florence Italy; ^4^ Fondazione Alitti O.N.L.U.S. Florence Italy; ^5^ Unit of Biostatistics Fondazione IRCCS Casa Sollievo della Sofferenza San Giovanni Rotondo Foggia Italy

**Keywords:** diabetes mellitus, diabetic foot, epidemiology, information sources, meta‐analysis

## Abstract

**Introduction:**

Diabetic foot (DF) disease, including ulcerations, amputations, and infections, represents a major cause of morbidity and mortality in individuals with diabetes mellitus (DM). Despite their substantial clinical, social, and economic burden, robust global epidemiological estimates remain limited due to fragmented evidence and heterogeneous methodologies. This systematic review and meta‐analysis aimed to provide reliable pooled estimates of incidence and prevalence of DF disease, to explore geographic and demographic variations, and to assess the types of data sources available for epidemiological research.

**Methods:**

Following PRISMA guidelines (PROSPERO registration CRD42025640944), PubMed, Embase, CINAHL Plus, and Cochrane Library were systematically searched from inception to December 21, 2024, for observational studies in adults (≥ 18 years) with DM reporting epidemiological measures of DF disease. Data extraction and quality appraisal (Joanna Briggs Institute checklists) were independently performed by four reviewers. Random‐effects meta‐analyses were conducted using generalised linear mixed‐effects models to calculate pooled complication‐specific prevalence and incidence rates among patients with diabetes. Heterogeneity among study estimates was assessed using Cochran's Q test, I^2^ statistic, and the between‐study variance (*τ* ^2^). To identify sources of heterogeneity, meta‐regression analyses were performed considering study‐level covariates (modifiers).

**Results:**

Eighty‐nine studies were included (18 prospective cohorts, 33 retrospective cohorts, 38 cross‐sectional), enrolling between 92 and 29,650,811 participants, with data from Europe (*N* = 29, 32.6%), Asia (*N* = 25, 28.1%), Africa (*N* = 16, 18.0%), America (*N* = 16, 18.0%), and Oceania (*N* = 3, 3.4%). Pooled global incidence rates were 10.93 cases/1000 person‐years (95% CI, 6.67–17.91) for ulcerations, 3.58 (95% CI, 1.93–6.63) for amputations, and 19.11 (95% CI, 4.81–75.97) for infections. Pooled prevalence estimates were 54.57 cases/1000 persons (95% CI, 37.63–78.52) for active ulcerations, 72.63 (95% CI, 48.21–108.02) for past ulcerations, 14.75 (95% CI, 9.52–22.77) for amputations, and 39.17 (95% CI, 6.81–195.07) for infections. Substantial heterogeneity (I^2^ > 90%) was observed, with significant modifiers including geographical region, diabetic foot identification method, data source, study setting, age, diabetes duration, and length of follow‐up. Rates in America and Africa were generally above the global pooled average, whereas those in Europe, Asia, and Oceania were below.

**Conclusions:**

Globally, DF disease is common and severe, with incidence and prevalence rates underscoring its important public health impact. Heterogeneity across studies highlighted the influence of geography, methodology, and data sources. These findings may support future clinical and pharmacoepidemiological studies that evaluate new interventions and monitor temporal trends using large‐scale data.

## 1 Introduction

Diabetic foot (DF) disease represents a major complication of diabetes mellitus (DM), substantially affecting the quality of life and increasing mortality among individuals with DM [1, 2, 3]. DF conditions include foot ulcers, lower‐limb amputations, and infections such as soft‐tissue infection and osteomyelitis, which together can result in a considerable burden on patients, their families, and healthcare systems [1, 2, 3, 4, 5, 6, 7]. These conditions typically arise from the combined effects of peripheral neuropathy, peripheral arterial disease, infection, and impaired wound healing, and represent major diabetes‐related causes of disability, healthcare use, and mortality, particularly when access to timely, specialised care is limited [2, 8].

Barriers to the prevention and early diagnosis of DF disease remain considerable and multifactorial. A lack of awareness among patients and caregivers often delays recognition of early warning signs, such as minor injuries or subtle changes in foot health [9, 10, 11]. Structured screening programs to detect early DM complications, including neuropathy and ischaemia, are frequently unavailable or inconsistently implemented, especially in low‐ and middle‐income countries where disparities in access to healthcare services exist or specialised DF services are scarce [12, 13, 14]. Moreover, healthcare professionals often receive insufficient training to effectively identify and manage DF conditions, while cultural and socioeconomic factors, such as stigma and the financial cost of care, may discourage patients from seeking timely medical attention [9, 10, 15]. The absence of advanced diagnostic tools and limited integration between primary care providers and specialists may further hinder the delivery of comprehensive, coordinated care [9, 16]. Addressing all these barriers could substantially reduce the burden of DF disease and improve outcomes for individuals with DM.

Understanding the scale and distribution of DF disease relies on robust epidemiological data. However, current estimates of DF prevalence and incidence are constrained by fragmented and heterogeneous evidence, and patterns across geographic regions and demographic groups remain poorly characterised [5, 12, 13, 17, 18, 19].

Several systematic reviews have previously summarised specific aspects of DF disease, including the global prevalence of DF ulceration, the incidence of diabetes‐related lower‐limb amputation, and the global burden of diabetes‐related lower‐extremity complications [17, 20, 21, 22, 23, 24, 25]. However, these reviews generally focused on individual outcomes, specific time periods, or burden‐of‐disease indicators. A comprehensive synthesis jointly examining multiple DF conditions, including ulceration, infection, and amputation, together with the methodological characteristics and data sources used to generate epidemiological estimates, remains limited. Addressing this information gap is critical for two main reasons: first, to inform public health initiatives and guide resource allocation and second, to support pharmacoepidemiological and pharmacovigilance research. Strong epidemiological evidence, together with a deeper understanding of methodologies and data sources employed in existing studies, could support the design of both targeted local investigations and large‐scale national studies [26, 27].

This systematic review and meta‐analysis aimed to address these knowledge gaps by providing reliable estimates of the global prevalence and incidence of DF disease among people with type 1 or type 2 diabetes, identifying factors influencing these estimates, and examining the data sources available for epidemiological research.

## 2 Methods

### 2.1 Search Strategy and Selection Criteria

This systematic review was carried out according to the Preferred Reporting Items for Systematic Reviews and Meta‐Analyses (PRISMA), and the study protocol was registered on PROSPERO (CRD42025640944). Observational studies reporting information on the epidemiology of DF disease were searched in the bibliographic databases PubMed (including PubMed Central and Medline), Embase, CINAHL Plus, and Cochrane Library from their inception until December 21, 2024. The search terms were related to DF and its consequences, as well as its epidemiology. The detailed search strategy developed for each database is provided in Supporting Information S1: Table S1.

Cohort (both prospective and retrospective) and cross‐sectional studies written in English that reported data on adults (≥ 18 years old) with DM (regardless of type) and epidemiological measures (e.g., prevalence, incidence) of DF conditions – such as ulcers, infections, ischaemia, and amputations [4]—were included in this systematic review. Narrative or systematic reviews and meta‐analyses, randomized controlled trials, case‐control studies, case reports, book chapters, editorials, and conference abstracts were excluded, but they were screened to identify other potentially relevant studies. Studies focusing exclusively on the general population without reporting epidemiological measures specific to the diabetic subpopulation, as well as studies exclusively addressing therapeutic or surgical interventions without providing epidemiological data, were excluded. After removing duplicates from the four databases, four review authors (M.C., F.M., D.P., and A.Fr.) individually screened titles and abstracts of the identified records to remove articles that were clearly irrelevant. The full texts of the selected articles were then independently reviewed to determine whether they met the inclusion criteria. Any disagreements among evaluators following both the title and abstract and the full‐text screening phases were resolved through discussion or, if consensus was not reached, through the intervention of a senior expert (G.T.).

### 2.2 Data Extraction

Data from each article included in the systematic review were independently extracted by four review authors (M.C., F.M., D.P., and A.Fr). Any discrepancy was resolved by consensus or, when necessary, through consultation with a senior expert (G.T.). For each article, the following information was extracted when available: first author, year of publication, country in which the study was conducted, data sources (e.g., claims databases, medical records, surveys), number of enroled participants, age and sex of the participants, characteristics of the study cohort, risk factors associated with the development of the DF disease, epidemiological measures (i.e., prevalence, incidence), clinical definitions of DF, and, for studies conducted in the general population, age, sex, and diabetes duration of the DF subpopulation. When reported, ulceration status was extracted according to the definitions used in the original studies. Active ulceration was defined as the presence of a diabetes‐related foot ulcer at the time of assessment, at the index date, or during the prevalence period defined by the study. Past or historical ulceration was considered as a previously reported history of diabetes‐related foot ulceration, in the absence of an active ulcer when this distinction was provided by the original study. No post hoc reclassification was applied beyond grouping comparable study‐specific definitions into active and past/historical ulceration categories.

### 2.3 Statistical Methods

Meta‐analyses of dichotomous outcomes were performed using generalised linear mixed‐effects models, fitting a random‐intercept logistic regression (binomial‐normal model) to estimate the pooled prevalence of DF disease (per 1000 persons with DM). Pooled logit‐transformed prevalence and standard error were obtained via maximum likelihood, with final estimates and 95% confidence intervals (CIs) derived using the inverse logit transformation. Between‐study heterogeneity was assessed using the Cochran's Q test, I^2^ statistic, and the between‐study variance (*τ* ^2^). Significance was defined as *p* < 0.10 or I^2^ > 40% [28].

For incidence rates (per 1000 person‐years with DM), a random‐intercept linear mixed‐effects model was used, assuming normality of the log‐transformed rates. The log‐normal random‐effects model was used to pool incidence rates, as most studies reported moderate‐to‐large event counts for which the normal approximation of log‐transformed incidence rates is generally adequate. Standard errors of the incidence rates were derived from the reported number of events, CIs, or *p*‐values. Specifically, the variance of the log‐incidence rate was approximated as 1/*n*. events when the number of events was available. When only incidence rates with confidence intervals or *p*‐values were reported, standard errors were reconstructed by inversion of the reported confidence limits or test statistics on the log scale.

In the presence of heterogeneity, meta‐regression was performed using predefined study‐level covariates (i.e., modifiers) to explain the between‐study variability. These covariates included the mean length of follow‐up (years), the study design (e.g., retrospective or prospective observational study or cross‐sectional study), the prevalence type (e.g., whether it was calculated during a specific year or time period), the population mean age at enrolment, the population mean diabetes duration, the mean percentage of women, the publication calendar year, the study period (defined as the year of the study start together with its duration), the DF identification method (i.e., diagnostic codes vs. others), the type of data source (e.g., claims databases, clinical registries, clinical visits, electronic or paper‐based health records), the population setting (e.g., population‐/registry‐based, primary/community care‐based, specialist outpatient clinic‐based, and hospital‐based/inpatient or tertiary care), and the geographical zone (e.g., America, Europe, Asia, Africa, and Oceania).

Covariate contributions were assessed via omnibus Wald‐type tests, and the proportion of reduction in total between‐study variance was expressed as *R* ^2^.

Forest plots displayed both individual and pooled estimates with 95% CIs, and studies were stratified by geographical zone. Funnel plots were used to assess publication bias, with asymmetry tested by Begg and Mazumdar's rank correlation (i.e., Kendall's *τ* coefficient). Meta‐regression, funnel plots, and publication bias tests were not performed when fewer than ten studies were included [29]. Statistical significance was set at two‐sided *p* < 0.05. All analyses were conducted using R (v4.4, metafor package).

### 2.4 Risk of Bias Assessment

The quality of the included studies was independently assessed by four review authors (M.C., F.M., D.P., and A.Fr.), using the Joanna Briggs Institute (JBI) critical appraisal checklists for prevalence and cohort studies, as appropriate [30]. Any discrepancy was resolved by consensus or, when necessary, through consultation with a senior expert (G.T.). The methodological quality of each study was rated based on the percentage of affirmative responses to the JBI checklists' items. Because JBI checklists do not prescribe fixed numerical thresholds for overall risk‐of‐bias categories, we applied predefined operational thresholds: studies with ≤ 49% positive responses were considered to have a high risk of bias, those with 50–74% a moderate risk, and those with ≥ 75% a low risk of bias. No studies were excluded based on the results of the quality appraisal.

## 3 Results

### 3.1 Study Selection

**FIGURE 1:**
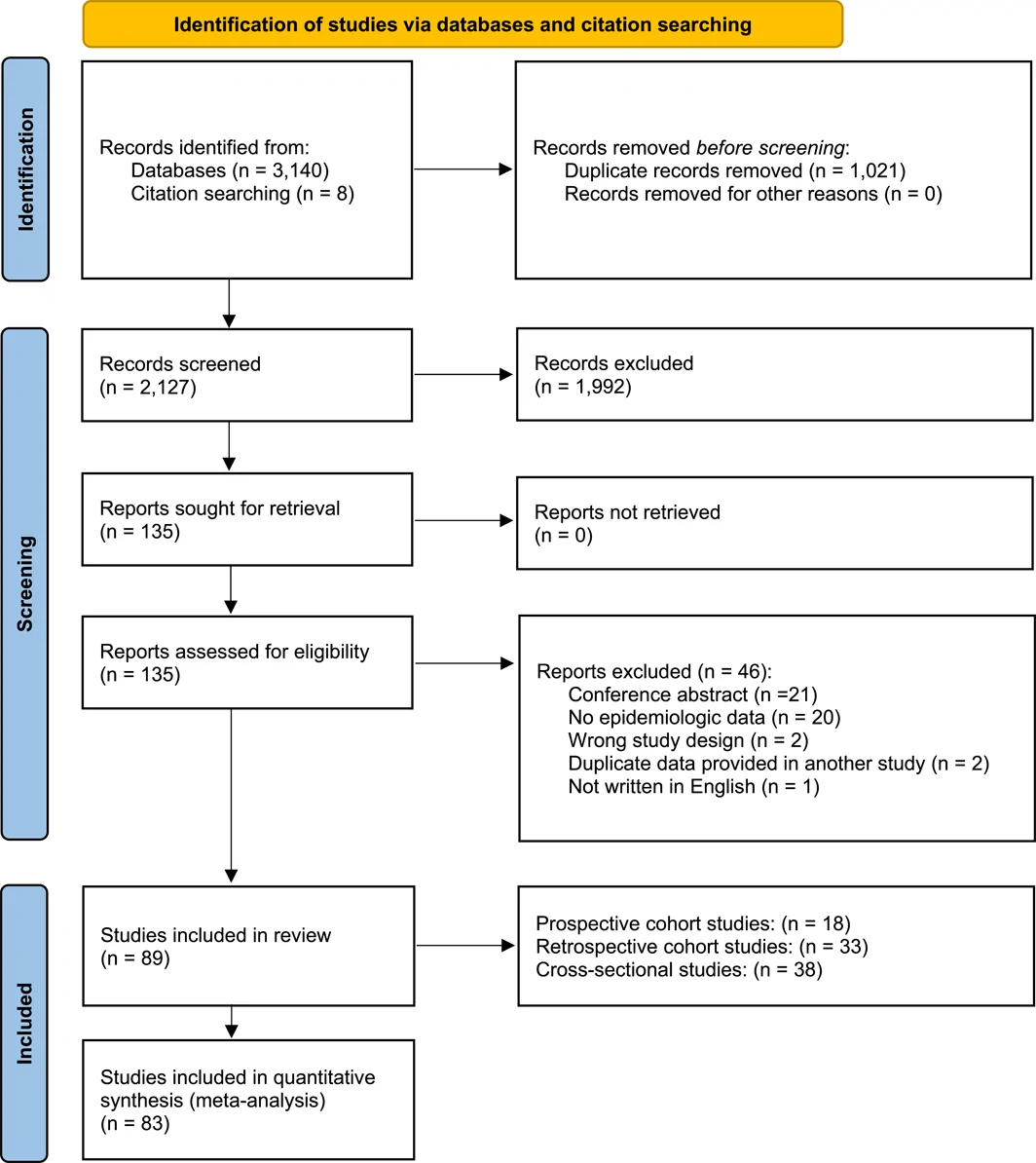
PRISMA flowchart showing the process of study selection.

A flowchart summarising the process of study selection is shown in Figure 1. Our search strategy identified a total of 3140 records, and 8 further records were identified through citation searching. After removing duplicate records (*N* = 1021), 2127 (67.6%) titles and abstracts were screened, and 135 (4.3%) full‐text articles were retained for further evaluation. Among these, 89 (65.9%) [18, 19, 31, 32, 33, 34, 35, 36, 37, 38, 39, 40, 41, 42, 43, 44, 45, 46, 47, 48, 49, 50, 51, 52, 53, 54, 55, 56, 57, 58, 59, 60, 61, 62, 63, 64, 65, 66, 67, 68, 69, 70, 71, 72, 73, 74, 75, 76, 77, 78, 79, 80, 81, 82, 83, 84, 85, 86, 87, 88, 89, 90, 91, 92, 93, 94, 95, 96, 97, 98, 99, 100, 101, 102, 103, 104, 105, 106, 107, 108, 109, 110, 111, 112, 113, 114, 115, 116, 117] met the inclusion criteria and were included in the systematic review (Figure 1). Reasons for the exclusion of the remaining full‐text articles are shown in Supporting Information S1: Table S2. Of the 89 studies included, 6 [48, 52, 55, 76, 85, 105] were excluded from the quantitative synthesis because they did not provide the necessary data (e.g., number of events or person‐years) to calculate standard errors or CIs for the reported rates or prevalence estimates. Specifically, one retrospective study [76] reported only age‐adjusted rates; three retrospective studies [48, 85, 105] reported incidence rates as point estimates without additional details; one retrospective study [55] presented the number of new cases observed over a 12‐year period relative to the baseline population; and one cross‐sectional study [52] provided prevalence percentages without absolute numbers.

### 3.2 Study Characteristics

The characteristics of the included studies are summarised in Table 1. Overall, 18 (20.2%) prospective cohort studies, 33 (37.1%) retrospective cohort studies, and 38 (42.7%) cross‐sectional studies were included in the systematic review. Twenty‐nine (32.6%) of them were conducted in Europe, while the others were conducted in Asia (*N* = 25, 28.1%), Africa (*N* = 16, 18.0%), America (*N* = 16, 18.0%), and Oceania (*N* = 3, 3.4%). More than half of these studies (*N* = 48, 53.9%) were published in the decade 2015–2024. The number of patients with DM enroled ranged from 92 [47] to 2,484,300 [45], and their mean age ranged from 44.3 [64] to 74.1 [69] years. Forty‐four studies (49.4%) included both patients with type 1 DM and patients with type 2 DM, 28 studies (31.5%) included only patients with type 2 DM, and 1 study (1.1%) included only patients with type 1 DM. The type of diabetes was not specified in 16 (18.0%) of the included studies.

**TABLE 1:** Characteristics of studies on diabetic foot disease included in the systematic review.

Author, year country	Type of data sources	Participants, *N*.	Age	Sex (women)	Diabetes duration	Type of diabetes	Study population details	Associated risk factors^a^	Epidemiological measures		Clinical definition of diabetic foot	Age, sex, and diabetes duration of the diabetic foot population
									Prevalence	Incidence		
Prospective cohort studies												
Nelson, 1988 [84], United States	Standardized physical and laboratory examination	4399	≥ 5 years	NR	NR	NR	Pima Indians from the general population	Male sex, duration of diabetes, retinopathy, nephropathy, medial arterial calcification, absence of patellar tendon reflexes, impaired vibration‐perception threshold of the great toes, high fasting and 2‐h post‐load plasma glucose concentrations	NR	Crude incidence: 10.4 per 1000 person‐years at risk (80 cases in 7723 person‐years at risk) in the 12‐year follow‐up period	Amputation	Age: 5–24 y: 0 (0.0%) 25–44 y: 17 (21.2%) 45–64 y: 38 (47.5%) ≥ 65 y: 25 (31.3%) F/M: 33/47 (41.2%/58.8%)
Moss, 1992 [75], United States	Standardized physical, laboratory, and instrumental examination	2990	Type 1, mean (SD): 29.3 (13.3) y Type 2, mean (SD): 54.8 (12.4) y	Type 1: 484 (48.6% of 996 analysed) Type 2: 736 (53.7% of 1370 analysed)	Type 1, mean (SD): 14.7 (10.6) y Type 2, mean (SD): 10.6 (8.2) y	Type 1: 1210 (40.5%) Type 2: 1780 (59.5%)	Diabetic patients receiving primary care. Type 1: Insulin‐taking individuals diagnosed before 30 years of age. Type 2: Random sample of people diagnosed at 30 years of age or older, regardless of insulin‐taking status	Type 1: HbA1c, retinopathy. In type 1 with onset ≥ 18 years of age: HbA1c, retinopathy, and smoking. Type 2: HbA1c, duration of diabetes, proteinuria, and diastolic blood pressure.	History of ulcer: Type 1: 96/879 (10.9%) Type 2: 98/955 (10.3%)	Type 1: 74/778 (9.5%) in the 4‐year follow‐up period Type 2: 90/856 (10.5%) in the 4‐year follow‐up period	Ulceration	F/M type 1: 392/386 (50.4%/49.6%) F/M type 2: 477/379 (55.7%/44.3%)
								Type 1: Age, history of sores/ulcers, diastolic blood pressure. In type 1 with onset ≥ 18 years of age: history of sores/ulcers, diastolic blood pressure, HbA1c, and smoking. Type 2: History of sores/ulcers, proteinuria, HbA1c, male sex, duration of diabetes.	Type 1: 24/996 cases (2.4%) Type 2: 60/1367 cases (4.4%)	Type 1: 19/879 (2.2%) in the 4‐year follow‐up period Type 2: 21/956 (2.2%) in the 4‐year follow‐up period	Amputation	Type 1: 0–29 y: 0 (0.0%) 30–39 y: 4/227 (1.8%) 40–49 y: 10/102 (9.8%) 50–59 y: 6/63 (9.5%) 60–69 y: 4/30 (13.3%) ≥ 70 y: 0 (0.0%) Type 2: < 40 y: 0 (0.0%) 40–49 y: 1/79 (1.3%) 50–59 y: 7/270 (2.6%) 60–69 y: 22/453 (4.9%) 70–79 y: 22/381 (5.8%) ≥ 80 y: 8/160 (5.0%) F/M type 1: 437/442 (49.7%/50.3%) F/M type 2: 533/423 (55.8%/44.2%) Diabetes duration Type 1: 0–4 y: 172 5–9 y: 246 10–14 y: 174 15–19: 134 20–24 y: 88 25–29 y: 81 39–34 y: 51 ≥ 35 y: 50 Type 2: 0–4 y: 353 5–9 y: 338 10–14 y: 180 15–19 y: 251 20–24 y: 160 25–29 y: 85 ≥ 30 y: 0
Rith‐Najarian, 1993 [95], United States	Diabetes registry, clinical visits, clinic amputation registry, insurance claims, death registry	346	0–24 y: 2 (0.6%) 25–34 y: 10 (2.9%) 35–44 y: 68 (19.7%) 45–54 y: 94 (27.2) 55–64 y: 98 (28.3%) > 64 y: 74 (21.4%)	182 (52.6)	NR	NR	Diabetic patients' residents in the North American Indian reservation community	NR	NR	26/1000 person‐years (27 cases in 1038 person‐years at risk)	Amputation	NR
Kästenbauer, 2001 [83], Austria	Clinical visits, health records, and death registry	187	Ulcerated subjects, mean (SD): 59.3 (7.6) y Non‐ulcerated subjects, mean (SD): 58.6 (8.0) y	85 (45.5%)	Ulcerated subjects, mean (SD): 11.2 (7.6) y Non‐ulcerated subjects, mean (SD): 10.5 (7.4) y	Type 2	Type 2 diabetes outpatient in a large referral centre, younger than 75 y. Past or current foot ulcers, lower‐extremity amputation, severe peripheral arterial disease (intermittent claudication), severe neurological deficits due to other diseases than diabetes, presence of any other cause of peripheral neuropathy, and Charcot's foot were exclusion criteria	BMI, alcohol intake, lower peroneal nerve conduction velocity, higher vibration perception threshold, and higher mean plantar pressure	NR	Annual incidence: 1.6% (95% CI 0.7–2.6) Incidence density: 21.7 (95% CI 8.2–35.1) per 1000 person‐years (10 cases)	Ulceration	Age, mean (SD): 59.3 (7.6) Diabetes duration, mean (SD): 11.2 (7.6) y
								NR	NR	6/187 cases for a mean follow‐up period of 3.6 years	Infection	NR
								NR	NR	4/187 cases for a mean follow‐up period of 3.6 years	Amputation	NR
Abbott, 2002 [31], United Kingdom	Clinical visits, paper‐based health records	9710	Mean (SD): 61.3 (14.1) y	4486 (46.2%)	Mean (SD): 8.9 (11.1) y	Type 1 and type 2	All adults diagnosed with type 1 or type 2 diabetes	History of foot ulcers (past or present at baseline), neuropathy, any previous podiatry or foot care advice, insensitivity to 10 g monofilament, reduced number of pedal pulses, foot deformities, increasing abnormal ankle reflex score, and age.	History of ulcer: 295 (3.1%) Active ulcer: 165 (1.7%)	Average annual incidence: 2.2% (291/6613 new foot ulcers in a follow‐up of 2 years)	Ulceration	NR
								NR	1.3% (*N*. = 122)	NR	Amputation	NR
Muller, 2002 [77], the Netherlands	Family physician's register, health records	1993: 552 1994: 579 1995: 614 1996: 655 1997: 682 1998:745	Mean age by year 1993: 67.4 1994: 67.5 1995: 67.7 1996: 67.6 1997: 67.2 1998: 67.1	1993: 62% 1994: 60% 1995: 59% 1996: 58% 1997: 58% 1998: 57%	Mean (range) 1993: 5.7 (0–28) y 1994: 6.0 (0–18) y 1995: 6.0 (0–22) y 1996: 6.3 (0–24) y 1997: 6.5 (0–21) y 1998: 6.4 (0–34) y	Type 2	All diabetic patients, including those under general practitioner and specialist care	Age, cardiovascular diseases, retinopathy, and one or more absent peripheral pulses	NR	Mean annual incidence: 2.1% (95% CI 1.52%–2.28%) (73 ulcers in 52 patients over the 6‐year study period)	Ulceration	Age, mean (range): 75.0 (54.0–94.0) F/M: 62%/38% Diabetes duration, mean (range): 8.9 (1.0–19.0) y
								NR	NR	Mean annual incidence: 0.6% (95% CI 0.37%–0.56%) (28 amputations in 15 patients over the 6‐year study period)	Amputation	Age, mean (range): 72.3 (59.0–90.0) F/M: 33%/67% Diabetes duration, mean (range): 10.3 (1.0–25.0) y
Boyko, 2006 [37], United States	Clinical visits, hospital medical records	1285	No incident ulcer, mean (SD): 62.4 (10.8) y Incident ulcer, mean (SD): 62.3 (9.2) y	2%	No incident ulcer, mean (SD): 10.0 (9.3) y Incident ulcer, mean (SD): 12.6 (10.0) y	All types, including type 2 diabetes (94.9%)	Ambulatory general internal medicine clinic patients with diabetes at a Veterans Affairs medical Centre. Exclusion criteria included a current foot ulcer and bilateral foot amputations	HbA1c, impaired vision, prior foot ulcer, prior amputation, monofilament insensitivity, tinea pedis, and onychomycosis	NR	Incidence: 5.0/100 person‐years (216 foot ulcers during a mean follow‐up duration of 3.38 years)	Ulceration	Age, mean (SD): 62.3 (9.2) F/M: 2%/98% Diabetes duration, mean (SD): 12.6 (10.0) y
Monami, 2009 [73], Italy	Standardized physical, laboratory, instrumental examination, hospital discharge records	1945	Mean SD: 64.0 (12.7) y	1103 (56.7%)	Mean (SD): 10.7 (10.5) y	Type 2	Type 2 diabetic outpatients referred to the diabetes clinic of a university hospital Geriatric Unit	Age, duration of diabetes, HbA1c, neuropathy, arteriopathy of the lower limbs, retinopathy, previous foot ulcer, and elevated pulse pressure	NR	Yearly incidence rate: 1.1% (86 ulcers during a mean follow‐up of 4.2 (± 2.2) years)	Ulceration	NR
Williams, 2010 [111], United States	Electronic/paper medical records, automated pharmacy, laboratory, and diagnosis data, diabetes registry, patient self‐reports, and questionnaires	3474	Mean SD: 64.1 (12.6) y	1667 (48.0%)	Mean (SD): 8.5 (8.2) y	Type 2	Type 2 diabetic patients from primary care clinics. Patients with prior diabetic foot ulcers and amputations were excluded	Major depression, peripheral arterial disease, peripheral neuropathy, and antidepressant use in the year prior to baseline.	NR	Unadjusted incidence: 8.4/1000 person‐years. Mean follow‐up: 4.1 years	Ulceration	NR
Crawford, 2011 [43], United Kingdom	Clinical visits, podiatry records, diabetes registry, telephonic interviews	1,192	Mean (SD): 70.53 (9.96) y	582 (48.8%)	Mean (SD): 8.88 (8.41) y	NR	Adult individuals with diabetes receiving podiatry care in community settings. Exclusion criterion was foot ulceration	Previous amputation, failure to obtain at least one blood pressure reading for the calculation of ankle‐brachial index with failure to feel touch with a 10‐g monofilament, use of insulin before 3 months with inability to distinguish between cool and cold temperatures	NR	Incidence: 1.93% (95% CI: 1.27–2.89) (23 patients with foot ulcers in an average 1‐year follow‐up).	Ulceration	Age, mean (SD): 72.09 (10.47) F/M: 5/18 (21.7%, 78.3%) Diabetes duration, mean (SD): 12.26 (8.47) y
Oe, 2014 [87], Japan	Medical records, examinator visits	578	Mean (SD): 65.4 (10.8) y	243 (42.0%)	Mean (SD): 13.8 (9.3) y	Type 1: 46 (8.0%) Type 2: 517 (89.4%) Others: 15 (2.6%)	Diabetic patients evaluated at a university hospital. Patients with foot ulcers at the start of the study were excluded	NR	NR	Cumulative incidence over the first 12 months: 0.2% (95% CI 0.03, 0.97), over 24 months: 0.4% (95% CI 0.09, 1.25), over 36 months: 0.8% (95% CI 0.27, 1.77), over 48 months: 1.0% (0.37, 2.01), and over 60 months: 1.2% (0.48, 2.25). Incidence: 0.21/1000 person‐month (6 cases in an average follow‐up period: 48.9 ± 16.0 months)	Ulceration	Age, median (IQR): 66 (60.3, 73.0) F/M: 4/2 (66.7%, 33,3%) Diabetes duration, median (IQR): 7 (4.3, 13.0) y
Robinson, 2015 [115], New Zealand	Cohort data linked with hospital records	71,777	Mean (SD) by ethnicity provided, i.e., European/other 66 (13), Māori 56 (12), Pacific 55 (12), South Asian 55 (12), and East Asian 59 (12) years	By ethnicity: 18,571 (48%) European/other, 4695 (52%) Māori, 5247 (57%) Pacific, 1256 (47%) south Asian, 1230 (51%) east Asian	Median (IQR) by ethnicity provided, i.e., European/other 4 (1–9), Māori 4 (1–9), Pacific 3 (1–8), south Asian 3 (1–8), and east Asian 3 (1–7) years	Type 2	Primary healthcare‐based cohort of diabetic patients, covering over half of New Zealand's diabetic population	Ethnicity, male sex, diabetes history (i.e., higher age of onset and duration), smoking, socio‐economic deprivation, higher height, systolic blood pressure (both high and low), higher HbA1c, and higher total/HDL cholesterol ratio	NR	892 amputations during 422,357 person‐years. Incidence rate: 2.11/1000 person‐years.	Amputation	NR
Park, 2016 [90], Republic of Korea	Cohort data and insurance claims	4087	< 50: 681 (16.7%) y 50–59: 1369 (33.5%) y 60–69: 1300 (31.8%) y ≥ 70: 737 (18.0%) y	Diabetic foot complication group: 255 (48.3%) Group without diabetic foot complications: 1505 (42.3%)	Diabetic foot complication group: Mean (SD) 10.16 (0.60) y Group without diabetic foot complications: Mean (SD) 9.78 (0.21) y	Type 2	Diabetic patients enroled across 12 hospitals. Ulcer at baseline was an exclusion criteria	Age, female sex, lower use of metformin, antiplatelet agents, higher HbA_1c_, and higher blood urea nitrogen	Active ulcer at baseline: 318 out of 4405 individuals (7.22%)	528 cases during 12,273 person‐years, with a median (95% CI) period of follow‐up of 3.30 years (1.39–4.48). Incidence rate: 43.02/1000 person‐years.	Diabetic foot	Age, mean (SD): 55.20 (0.87) F/M: 255/273 (48.3%, 51.7%) Diabetes duration, mean (SD): 10.16 (0.60) y
Yun, 2016 [117], South Korea	Clinical visits and laboratory analyses	595	Mean (SD): 53.5 (9.7) y	271 (60.4%)	Mean (SD): 6.1 (5.2) y	Type 2	Outpatients (from a university‐affiliated diabetes centre) with type 2 diabetes between the ages of 25 and 75 years. Exclusion criteria: Diabetic polyneuropathy, presence of arrhythmia or severe illness, chronic kidney disease stage 3 and higher, former or current foot ulcer, and previous amputation	Cardiovascular autonomic neuropathy, HbA1c, and diabetic nephropathy	NR	22/449 for a median follow‐up time of 13.3 y Incidence: 3.7 per 1000 patient‐years	Ulceration	Age, mean (SD): 55.6 (11.4) Sex: M 9 (40.9%), F 13 (59.1) Diabetes duration, mean (SD): 8.5 (6.6) y
								NR	NR	6/449 for a median follow‐up time of 13.3 y	Amputation	NR
Iwase, 2018 [59], Japan	Medical records, self‐administered questionnaire	4870	Mean: 65 y	2115 (43.4%)	Patients with ulcer, mean (SD): 19.5 (9.7) y Patients without ulcer, mean (SD): 15.5 (10.5) y	Type 2	Patients with type 2 diabetes attending teaching hospitals and certified diabetes clinics. Exclusion criteria for the registry are: (1) patients with drug‐induced diabetes or those undergoing corticosteroid treatment; (2) patients undergoing renal replacement therapy; (3) patients with serious diseases other than diabetes, such as advanced malignancies or decompensated liver cirrhosis; and (4) patients unable to visit a diabetologist regularly	Longer duration of diabetes, less leisure‐time physical activity, higher prevalence of depressive symptoms, poor glycaemic control, insulin therapy, antihypertensive use, eGFR < 60 mL/min, eGFR < 30 mL/min, history of diabetic foot ulcer, history of limb amputation, peripheral arterial disease, and cardiovascular disease, and lower LDL cholesterol	History of ulcer: 98/4870 (2.01%)	Incidence rate: 2.9/1000 person‐years (74 cases in a median follow‐up period of 5.4 years) Incidence: 0.3% per year	Ulceration	Age, mean (SD): 67.1 (9.4) F/M: 29/45 (39.2%/60.8%) Diabetes duration, mean (SD): 19.5 (9.7) y
								NR	History of amputation: 22/4870 (0.45%)	Incidence rate: 0.47/1000 person‐years (12 cases in a median follow‐up period of 5.4 years) Incidence: 0.05% per year	Amputation	NR
Panagoulias, 2020 [89], Bulgaria, Greece, Serbia, and United Kingdom	Medical records and physical exam	308	Patients with ulcer, mean (SD): 65.7 (11.2) Patients without ulcer, mean (SD): 62.0 (1.3) y	155 (50.3%)	Patients with ulcer, median (IQR): 15 (4–23) y Patients without ulcer, median (IQR): 10 (4–17) y	Type 1: 20 (6.5%) Type 2: 288 (93.5%)	Diabetic patients who attended the outpatient diabetes clinics of the participating hospitals. Exclusion criteria were patients having a history of previous ulceration/amputation, Charcot foot, critical limb ischaemia, stroke, causes of neuropathy other than diabetes, severe liver or kidney diseases, current or history of malignancies, uncontrolled thyroid disease, dermatological diseases that can cause dry skin, and recent local application of hydrating lotions, creams, and gels	Age, longer diabetes duration, neuropathy, retinopathy, and coronary artery disease	NR	Annual incidence: 2.97% Overall incidence: 17.85% (55 cases during the 6‐year follow‐up period)	Ulceration	Age, mean (SD): 65.7 (11.2) F/M: 23/32 (41.8%/58.2%) Diabetes duration, median (IQR): 15 (4–23) y Type 1/type 2, N.: 3/52 (5.5%/94.5%) y
								NR	NR	Annual incidence: 0.37% Overall incidence: 2.27% (7 cases during the 6‐year follow‐up period)	Amputation	NR
Bundó, 2021 [39], Spain	Medical records and physical exam	47,227	Mean (SD): 72.2 (12.7) y	78 (30.5%)	Mean (SD): 13.5 (8.1) y	Type 2	Adult patients with diabetes attending a primary care centre	NR	NR	Annual cumulative incidence: 0.42%. Cumulative incidence: 4.17/1000 person‐years. (256 cases during the 18‐month study period)	Ulceration	NR
Yazdanpanah, 2024 [112], Iran	Checklist completed by study participants, diagnostic tests performed by healthcare professionals, and telephonic interviews	901	Mean (SD): 53.24 (11.46) y	525 (58.3%)	Mean (SD): 9.4 (6.8) y	Type 1 and type 2	Diabetic patients referring to a diabetic foot clinic	History of diabetic foot ulcer or amputation, ill‐fitting footwear, absence of preventive foot care, and smoking	NR	The 2‐year cumulative incidence of diabetic foot ulcer was 8% (95% CI 0.071, 0.089) (72 cases). Incidence rate: 49.9/1000 person‐years	Ulceration	Age, mean (SD): 53.94 (12.92) F/M: 38/34 (52.8%/47.2%) Diabetes duration, mean (SD): 9.51 (6.00) y
Retrospective cohort studies												
Most, 1983 [76], United States	Discharge data from hospital abstracting services	NR	NR	NR	NR	NR	Individuals with diabetes	Age, male sex, and African American origin	NR	Estimated age‐adjusted rate: 59.7/10,000	Amputation	NR
Lindegard, 1984 [69], Sweden	Register of the operation ward and county population registry	Gotland (1980): 54,475 Umeå (1980): 115,497	Mean (Gotland): 73.9 y Mean (Umeå): 74.2 y	Gotland: 59% Umeå: 52%	Mean (Gotland): 8.8 Mean (Umeå): 12.0 y	NR	General population	NR	NR	Gotland: 20.5/100,000 inhabitants per year (111 cases during 1971–1980) Umeå: 6.5/100,000 inhabitants per year (71 cases during 1971–1980)	Amputation	NR
Waugh, 1988 [109], United Kingdom	Hospital discharge records, specialist clinic records, death register, questionnaires to general practitioners	3062	< 65 y: 55% > 65 y: 45%	NR	NR	NR	Patients with diabetes from the general population	Male sex	NR	156/3062 patients in 1981–1982	Amputation	Age, mean (SD): F: 74.7 (1.9) y M: 66.3 (2.3) y F/M: 61/95 (39.1%/60.9%)
Ebskov, 1991 [48], Denmark	Amputation registry, hospital discharge records	NR	NR	NR	NR	Type 1 and type 2	Patients with diabetes from the general population	NR	NR	Rate first amputation: 601/100,000 (1980) 376/100,000 (1989) (From 1980 to 1989: 5642 amputations on 4852 patients)	Amputation	Age, mean: 69.9 y (M), 73 y (F) M To F ratio: 1:0.9 Type 1: 30% Type 2: 70%
Deerochanawong, 1992 [44], United Kingdom	Surgery operation records, hospital discharge records, medical records	279,260	NR	NR	NR	NR	General population	Peripheral vascular disease, peripheral neuropathy, foot ulcer, previous history of lower limb amputation, and smoking	NR	5.7 per 100,000 inhabitants per year (48/279,260 during the 3‐year study period) 5.7 per 1000 diabetic patients per year (considering 1.0% the prevalence of people with diabetes)	Amputation	Age, mean (range): 71 (54–85) y for men Mean (range): 67 (27–88) y for women F/M: 12/36 (25%/75%) Duration of diabetes, mean (range): 7 (0–27) y
Farrell, 1993 [51], United States	Electronic health records, health summaries generated from written assessments documented by physicians, clinical registries	606	35–44 y: 119 (19.1%) 45–54 y: 132 (21.7%) 55–64 y: 138 (29.7%) 65+ y: 165 (29.7%) (52 unknown)	NR	NR	Type 1 and type 2	American Indians with type 1 or type 2 diabetes	NR	NR	Annual age‐adjusted first amputation rate: 121/10,000 Annual age‐adjusted all amputation rate: 181/10,000 (from 1982 to 1989: 66 amputations in 44 patients)	Amputation	NR
Lee, 1993 [65], United States	Medical records, follow‐up examination	1011	Mean (SD): 51.6 (10.8) y	632 (62.5%)	Mean (SD): 6.6 (6.1) y	Type 2	Indians with non‐insulin‐dependent diabetes mellitus	Retinopathy, insulin use, blood pressure, duration of diabetes, fasting plasma glucose, plasma cholesterol	At baseline: 21/1011	10‐y Cumulative incidence: 156/875 cases (17.8%) First amputation incidence rate: 18.0/1000 person‐years (95% Cl 10.6–29.8)	Amputation	F/M: 75/81 (48.1%/51.9%)
Valway, 1993 [105], United States	Hospital discharge records, ambulatory patient care reporting system	929	NR	421 (45.3%)	NR	Type 1 and type 2	American Indians with diabetes from the general population	Age	NR	Annual incidence: < 15 y: 0/10,000 15–44 y: 48.1/10,000 45–64 y: 85.9/10,000 65+ y: 104.1/10,000	Amputation	Age < 15 y: 0 (0.0%) 15–44 y: 146 (15.7%) 45–64 y: 478 (51.5%) 65+ y: 305 (32.8%)
New, 1997 [85], United Kingdom	Diabetes registry	4941	< 45: 747 y (15.1%) 45–64: 1886 y (38.2%) 65–74: 1324 y (26.8%) > 75: 984 y (19.9%)	2377 (48.1%)	NR	Type 1: 837 (16.9%) Type 2: 4104 (83.1%)	Individuals with newly diagnosed diabetes	Male sex, smoking, peripheral vascular disease, neuropathy, ischaemic ulcer, neuropathic ulcer, ulcers on neuropathy without peripheral vascular disease	NR	10.2 per 100,000 population per year 475 per 100,000 diabetic patient‐years (94 amputations in 79 patients during the 5‐y study period)	Amputation	Age, mean (SD): 62 (12) y Type 1: 17 (21.5%) Type 2: 62 (78.5%) F/M: 20/59 (25.3%/74.7%) Diabetes duration < 5 y: 28 (35.4%) > 5 y: 51 (64.6%)
Lavery, 1999 [62], United States	Medical records, operative reports, physicians/nurses admission notes	NR	NR	NR	NR	NR	Mexican Americans, African Americans, and non‐Hispanic whites with diabetes	Previous amputation, male sex	NR	Male: 133.19/10,000 (95% CI 117.39–148.99) Female: 56.57/10,000 (95% CI 49.39–63.75) (1944 amputations occurred during 1228 hospital episodes in 1043 subjects)	Amputation	Age, mean (SD): 64.8 (12.5) y F/M: 427/616 (40.9%/59.1%)
Ramsey, 1999 [93], United States	Decision support system linking clinical and financial data from several departments (medical staff, nursing, pharmacy, laboratory, radiology, hospital inpatient, and community health services)	8905	Mean (SD): 65.8 (12.5) y	4274 (48%)	NR	Type 1 and type 2	Patients aged 18 years and older with type 1 or type 2 diabetes. Patients with a diagnosis of osteomyelitis or a lower‐extremity amputation at any time prior to the date of foot ulcer diagnosis were excluded from the analysis.	NR	NR	514/8905 cases during the 3‐y of observation (1.9% per year, cumulative incidence 5.8% over 3 years)	Ulceration	Age, mean (SD): 66.3 (12.8) y M/F: 247/267 (52%/48%)
Holstein, 2000 [58], Denmark	Hospital clinical records	1981: 178,501 1995: 174,892	1981: < 45 y: 87,068 (48.8%) 45–69 y: 58,703 (32.9%) > 70+ y: 32,730 (18.3%) 1995: < 45 y: 106,845 (61.1%) 45–69 y: 39,318 (22.5%) > 70+ y: 28,729 (16.4%)	NR	NR	Type 1 and type 2	General population	NR	NR	463 cases during the 15‐y follow‐up period. Total 1981: 27.2/100,000 1995: 6.9/100,000 Type 1 DM 1981: 10.0/100,000 1995: 4.1/100,000 Type 2 DM 1981: 17.2/100,000 1995: 2.8/100,000.	Amputation	Type 1: M 82, F 65 Age, mean (range): M 66.3 (47–85) y, F 72.4 (45–90) y Type 2: M 188, F128 Age, mean (range): M 71.7 (56–88) y, F 75.2 (59–93) y
Morgan, 2000 [74], United Kingdom	General practice audit database and a hospital‐based record linkage‐derived patient index	10,287	> 15 y	NR	NR	Type 1: 16.1% Type 2: 83.9%	Hospital and general practice patients with type 1 or type 2 diabetes	NR	1865/10,287 (18.1%) during the 6‐y study period		Diabetic foot	NR
Lavery, 2003 [63], United States	Administrative databases, medical records, laboratory data, and communication with the primary care physician	1666	Mean (SD): 69.1 (11.1) y	828 (49.7%)	Mean (SD): 11.2 (9.5) y	Type 1 and type 2	Mexican Americans and non‐Hispanic whites with diabetes in a disease management programme	NR	History of ulcer at baseline: 122 (7.3%)	68.4/1000 person‐years	Ulceration	NR
								NR	NR	36.5/1000 person‐years	Infection	NR
								NR	At baseline: 58 (3.5%)	5.9/1000 person‐years	Amputation	NR
Schofield, 2009 [101], United Kingdom	Diabetes register, hospital discharge database, medical records, regional biochemistry database, and the community‐based mobile diabetic eye screening facility	NR	NR	NR	NR	NR	Patients with diabetes from the general population	NR	NR	397 amputations during the 7‐y study period. 2000: 5.1/1000 (95% CI 3.8–6.4) 2006: 2.9/1000 (95% CI 1.9–3.8)	Amputation	Age, mean (range): 68.1 (28–97) y F/M: 121/276 (30.5%/69.5%) Diabetes duration, mean (range): 12.1 (0–59.8) y
Bruun, 2013 [38], Denmark	Cohort data, nationwide administrative health registry (including hospital discharge data and outpatient clinics data), and mental health administrative registry	1381	With ulcer, median (IQR): 64.4 (56.9–73.1) y Without ulcer, median (IQR): 65.4 (55.6–73.6) y	NR	NR	Type 2	Patients with a newly diagnosed diabetes, aged 40 y or older	Peripheral neuropathy, peripheral arterial disease, male sex, retinopathy, and myocardial infarction	Point prevalence of foot ulcer: At diagnosis: 2.76% (95% CI 1.89–3.63) At 6 years: 2.93% (95% CI 1.86–4.00) At 14 years: 4.96% (95% CI 3.10–6.82)	88/1381 cases over the 14‐y follow‐up period	Ulceration	At 14‐year of follow‐up: Age, median (IQR): 73.2 (65.4–81.6) y F/M: 13/13 (50%/50%) Diabetes duration, median (IQR): 14.0 (13.3–14.5) y
								Male sex, myocardial infarction, peripheral neuropathy, peripheral arterial disease, microalbuminuria, retinopathy, and impaired vision or blindness	NR	63/1381 cases over the 14‐y follow‐up period (15,754 years at risk) Incidence: 400 (95% CI 307–512) per 100,000 patient‐years	Amputation	Age, median (IQR): 62.6 (54.5–70.0) y F/M: 20/43 (31.8%/68.2%)
Jørgensen, 2013 [92], Denmark	Electronic medical record system of the diabetes centre, nationwide administrative patient registry (including hospital discharge data and outpatient clinics' data)	11,332	Median, (IQR): Type 1: 44 (32–57) y Type 2: 60 (51–75) y	NR	Median (IQR) Type 1: 19 (8–29) y Type 2: 9 (4–14) y	Type 1: 5116 (45.1%) Type 2: 6216 (54.9%)	Diabetic patients followed at a specialist centre	Male sex, diabetes duration	NR	384 cases (2000–2011) during 100,495 patient‐years of follow‐up	Amputation	NR
Chang, 2015 [41], Taiwan	Hospital medical records and laboratory analyses	362	Mean (SD): Group 1 (*N*. = 191): 67.3 (8.1) y Group 2 (*N*. = 20): 69.2 (8.9) y Group 3 (*N*. = 125): 67.4 (9.4) y Group 4 (*N*. = 26): 68.5 (8.2) y	166 (45.9%)	Mean: 4.8 y	Type 2	Inpatients with type 2 diabetes. ABI > 1.3 was an exclusion criteria	NR	NR	9/362 during the 2.5 y follow‐up period	Diabetes foot (ulceration or infection)	NR
								NR	NR	1/362 during the 2.5 y follow‐up period	Amputation	
Tomita, 2016 [102], Japan	Hospital and outpatient clinics medical records	1305	Mean (SD): 59.9 (9.1) y	401 (30.7%)	Mean (SD): 10.3 (8.2) y	Type 2	Patients with type 2 diabetes who had undergone a 2‐week education programme for diabetic patients. Exclusion criteria: The presence of an active diabetic foot ulcer at the time of inclusion, an estimated glomerular filtration rate (eGFR) < 30 mL/min/1.73 m^2^ or a strong suspicion of renal disease other than diabetic nephropathy and a follow‐up period < 1 year	Albuminuria, diabetic retinopathy, angiopathy, and neuropathy	NR	50 cases during the median follow‐up of 11.8 y Time at risk (person‐ years): 14,291 Incidence: 3.5 per 1000 person‐years Cumulative incidence: 5‐Year: 1.9% (95% CI 1.1–3.3) 10‐year: 3.7% (95% CI 2.7–5.0)	Ulceration	NR
Anderson, 2017 [35], United Kingdom	Primary care electronic health records (including 42 general practices)	13,955	Mean: 69.4 (range: 16–89) y	6011 (43.1%)	NR	Type1: 1370 (9.8%) Type2: 12,585 (90.2%)	Individuals with type 1 or type 2 diabetes, with no prior history of foot ulceration	Age, hypertension, cerebrovascular disease, peripheral vascular disease, previous myocardial infarction, congestive cardiac failure, renal function, higher HbA_1c_, cholesterol and statin treatment, current/ex‐smoker, and social disadvantage (Townsend index)	NR	Overall: 1147 (8.2%) Type 1: 1081 (4.8%) Type 2: 66 (8.6%) (Median follow‐up: 10.5 years)	Ulceration	Type 1 Age, mean (95% CI): 59.33 (55.3–63.4) y F/M: 21/45 (31.8%/68.2%) Type 2 Age, mean (95% CI): 74.2 (73.5–74.9) y F/M: 487/594 (45.1%/54.9%)
Rasmussen, 2017 [94], Denmark	Electronic patient record system of a large specialised diabetes hospital	12,680	< 40 y: Type 1: 2593 (46.5%) Type 2: 513 (7.2%) 40–59 y: Type 1: 2109 (37.8%) Type 2: 2912 (41.0%) 60–79 y: Type 1: 805 (14.4%) Type 2: 3324 (46.8%) ≥ 80 y: Type 1: 75 (1.3%) Type 2: 349 (4.9%)	Type 1: 2534 (45.4%) Type 2: 2901 (40.9%)	< 5 years: Type 1: 1118 (20.2%) Type 2: 2707 (38.9%) 5–20 years: Type 1: 2214 (40.0%) Type 2: 3787 (54.4%) > 20 years: Type 1: 2199 (39.8%) Type 2: 472 (6.8%)	Type 1: 5582 (44.0%) Type 2: 7098 (56.0%)	Diabetic patients referred to a specialised diabetes hospital, without history of foot ulcer	NR	History of ulcer at baseline: Type 1: 38 out of 5678 (0.67%) Type 2: 49 out of 7002 (0.70%)	255 out of 5640 patients with type 1 diabetes developed an ulcer. Crude incidence (95% CI): 5.8 (5.1–6.5)/1000 patient‐years. Person‐years = 44,279 310 out of 6953 patients with type 2 diabetes developed an ulcer. Crude incidence (95% CI): 11.3 (10.1–12.6)/1000 patient‐years. Person‐years = 27,491	Ulceration	NR
Heald, 2019 [55], United Kingdom	Electronic health records	17,053	Mean (SD): Cases: 77.9 (14.1) Non cases: 73.8 (16.9)	7682 (45.0%)	NR	NR	All men and women aged 16–89 years, attending general practices. History of foot ulceration was an exclusion criteria	Age, HbA1c, absence of monofilament sensation, creatinine level, and history of stroke	NR	1127/17,053 cases over the 12‐y follow‐up period	Ulceration	Age, mean (SD): 77.9 (14.1) F/M: 522/605 (46.32%/53.68%)
Paisey, 2019 [88], United Kingdom	Community podiatry database and clinical examination	1640	NR	NR	NR	NR	Individuals from community podiatry (people with high risk of diabetes related foot ulceration and those with new foot ulcers)	NR	History of ulcer and active ulcer combined: 2003–2007: 20.7/1000 persons 2008–2012: 24.9/1000 persons 2013–2017: 33.1/1000 persons	1640 cases during the 18‐y period 2003–2007: 11.1/1000 person‐years 2008–2012: 12.5/1000 person‐years 2013–2017: 6.1/1000 persons‐years	Ulceration	Age mean (SD): 2003–2007 73.1 (12.2); 2008–2012 74.0 (12.0); 2013–2017 76.2 (12.2) Sex F: 2003–2007: 44.5% 2008–2012: 39.9% 2013–2017: 37.1%
Adem, 2020 [33], Ethiopia	Hospital medical records	387	Median (IQR): 46 (35–58) y	154 (39.8%)	< 5 years: 256 (66.15% ≥ 5 years: 131 (33.85%)	Type 1: 131 (33.85%) Type 2: 256 (66.15%)	Patients with a new diagnosis of diabetes. Exclusion criteria was foot ulcer at diabetes diagnosis	Increased BMI, diabetic retinopathy, and diabetic nephropathy	NR	Incidence: 4.00 (95% CI 3.13–5.05) per 100 person‐years [66 cases, follow‐up time: median (range): 95 (4–120) months]	Ulceration	Age, median: 46 y Type 1: 10 (15.2%) Type 2: 56 (84.8%)
Chou, 2020 [42], Taiwan	Population‐based health insurance database, death registry	123,172	Mean (SD): 60.7 (12.4) y	58,276 (47.3%)	0 (analysis conducted at disease onset)	Type 1 and type 2	Individuals with newly‐diagnosed diabetes	Diabetes onset at older ages	At baseline: 1075 (0.873%)	NR	Infection or amputation	NR
									At baseline: 1056 (0.857%)	NR	Infection	NR
									At baseline: 85 (0.069%)	NR	Amputation	NR
Déruaz‐Luyet, 2020 [45], United States	Commercial insurance claims database	29,650,811	NR	Type 1: 46.5% Type 2: 46.7%	NR	Type 1: 183,889 (7.4%) Type 2: 2,300,411 (92.6%)	General population. Total patient with diabetes: 2,484,300. Patients with type 1 or type 2 diabetes aged 18 or older were included. Prior traumatic amputation was an exclusion criteria	NR	NR	Type 1: 5.79/1000 person‐years (2366 patients per 408,328 total person‐years) Type 2: 1.62/1000 person‐years (9222 patients per 5,691,794 total person‐years)	Amputation	Type 1 Age: < 65 y: 1557 (65.8%) > 65 y: 809 (34.2%) F: 760 (32.1%) Type 2 Age: < 65 y: 5626 (61.0%) > 65 y: 3596 (39.0%) F: 2739 (29,7%)
Li, 2020 [67], Taiwan	Diabetes registry, hospital medical records	Derivation set: 21,484	Mean (SD): 61.02 (10.81) y	11,518 (53.61%)	Mean (SD): 6.54 (6.29) y	Type 2	Patients with type 2 diabetes without prior amputation, aged 30–85 years old	NR	At baseline: 85/32,226 (0.26%)	NR	Ulceration	NR
		Validation set: 10,742	Mean (SD): 61.05 (10.8) y	5734 (53.38%)	Mean (SD): 6.53 (6.40) y			Age, gender, duration of type 2 diabetes, body mass index, HbA1c, triglyceride, eGFR, variation of fasting blood glucose, comorbidities of stroke, diabetes retinopathy, hypoglycemia and foot ulcer, anti‐diabetes medication, and use of diuretics and nitrates	NR	504/32,226 patients at an average follow‐up of 7.4 y	Amputation	NR
Uivaraseanu, 2020 [103], Romania	Hospital medical records	2992	NR	NR	NR	Type 1 and type 2	Inpatients with type 1 or type 2 diabetes	Age, HbA1c, diabetes duration, diabetic retinopathy, chronic kidney disease, diabetic polyneuropathy, peripheral artery disease, and coronary artery disease	Two‐year period prevalence: 252/2992 (8.42%)	NR	Ulceration	Age, mean (SD): 65.72 (11.89) y F/M: 101/151 (40.08%/59.92%) Type 1: 17 (6.7%) Type 2: 235 (93.3%) Diabetes duration, mean (SD): 11.8 (7.8) y
Lu, 2021 [71], Taiwan	Population‐based health insurance database	340,786 (Pay‐for‐Performance‐P4P group) versus 851,382 (Non‐P4P group), after matching 312,962 in each group	After matching: Mean (SD) P4P group: 60.7 (11.9) y Mean (SD) non‐P4P group: 60.7 (13.1) y	After matching: P4P group: 157,107 (50.2%) Non‐P4P group: 156,385 (50.0%)	After matching: Mean (SD) P4P group: 5.4 (4.0) y Mean (SD) non‐P4P group: 5.3 (4.1) y	Type 2	Type 2 diabetic patients, without prior diabetic foot	NR	NR	P4P group: 6185 (2.0%)^b^ Non‐P4P group: 8022 (2.6%)^b^	Major adverse limb events, including ulceration, amputation, gangrene, and surgery (angioplasty)	NR
										P4P group: 3483 (1.1%)^b^ Non‐P4P group: 4167 (1.3%)^b^	Ulceration	NR
										P4P group: 1439 (0.46%)^b^ Non‐P4P group: 2349 (0.75%)^b^	Amputation	NR
	
Rossboth, 2021 [116], Austria	Hospital medical records and medical record from outpatient practices of specialists of internal medicine	10,688	Mean (SD): 63.21 (12.58) y	44.3%	0 (analysis conducted at disease onset)	Type 2	Inpatients and outpatients with type 2 diabetes mellitus aged ≥ 18 years from diagnosis, without diabetic foot or prior diabetic foot at baseline	Male gender, nephropathy, neuropathy, peripheral arterial disease, and coronary bypass. Increased age at diabetes diagnosis and use of insulin or insulin analogues was associated with a lower likelihood of diabetic foot events	NR	140/10,688 cases (1.31%) during a mean follow‐up of 9.75 y	Ulceration	Age, mean (SD): 63.0 (11.9) y F/M: 42/98 (30.0%/70.0%)
Tai, 2021 [97], Taiwan	Population‐based health insurance database	2001: 61,593 2015: 162,873	NR	NR	NR	Type 1 and type 2	Patients with type 1 and type 2 diabetes from the general population	NR	Annual prevalence: 2001: 325/61,593 cases (0.53%) 2015: 849/162,873 cases (0.52%) (7511 new patients with ulcer from 2001 to 2015)	NR	Ulceration	Age, mean (SD): 63.2 (13.5) y F/M: 3317/4194 (44.2%/55.8%)
Lovell, 2022 [70], Barbados	Electronic medical records	225	Mean (SD): 56.8 (13.4) y	159 (70.7%)	NR	Type 1: 9 (4%) Type 2: 216 (96%)	Adult (> 18 y) patients with type 1 or type 2 diabetes attending a publicly funded programme for persons with a new diagnosis or poorly controlled type 2 diabetes. Exclusions criteria: Pregnancy/lactation and persons with traumatic/post‐surgical lower extremity amputations	Age, hypertension, retinopathy, chronic kidney disease, peripheral nephropathy, clopidogrel use, and aspirin use.	1‐y period prevalence: 33/225 (14.7%; CI: 10.5, 20.1)	4.4% (95% CI 4.4, 4.5)	Ulceration	Age, mean (SD): 61.7 (10.7) y F/M: 20/13 (60.6%/39.4%)
Negussie, 2024 [82], Ethiopia	Hospital medical records	418	Median (IQR): 47 (35–58) y	195 (46.65%)	< 5 years: 263 patients (62.92) ≥ 5 years 155 patients (37.08)	Type 1: 76 (18.18%) Type 2: 342 (81.82%)	Inpatients and outpatients with diabetes from a hospital medical college. Patients with foot ulcer at diabetes diagnosis were excluded	Diastolic blood pressure of 90 mmHg or above, taking both oral hypoglycemic agents and insulin, and peripheral arterial disease	NR	Cumulative incidence: 26 cases per 1716.48 person‐years (6.2%; 95% CI 4.1%–8.6%) Overall incidence rate: 1.51/100 person‐years (95% CI 1.03–2.22)	Ulceration	NR
Cross‐sectional studies												
Walters, 1992 [108], United Kingdom	Cohort data, diabetic registers held by the GPs, medical records from the hospital diabetic clinics, and repeat prescription requests and letters in each practice, collected by the GPs' receptionists	1150	NR	NR	NR	Type 1: 19.7% Type 2: 80.3%	Patients with diabetes within 10 general practices	For ulceration: Duration of diabetes, absent light touch, impaired pain perception, absent dorsalis pedis pulse, and any retinopathy	History of ulcer and active ulcer combined: Overall: 7.4 (95% CI 5.8–9.0) % Type 1 diabetes: 7.7 (95% CI 4.1–11.3) % Type 2 diabetes: 6.8 (95% CI 5.1–8.5) % Active ulcer: 3.3% (95% CI 2.2–4.4) in diabetic patients aged 30 years or more	NR	Ulceration	NR
									1.3 (95% CI 0.6–2.0) % in diabetic patients (14 cases, 3 with type 1 diabetes and 11 with type 2 diabetes)	NR	Amputation	NR
Nambuya, 1996 [79], Uganda	Record sheet, hospital records	252	Mean (range): 45 (30–69) y	135 (53.6%)	0 (analysis conducted at disease onset)	NR	Newly diagnosed diabetic patients from a hospital diabetic clinic	NR	Active ulcer: 10 (4.0%)	NR	Ulceration	F/M: 4/6 (40%/60%)
Gulliford, 2002 [54], Trinidad and Tobago	Questionnaires and laboratory analyses from medical records	2106	With previous foot ulcer, median (IQR): 61 (52–67) y Without previous foot ulcer, median (IQR): 60 (52–67) y	1478 (70.2%)	With previous foot ulcer, median (IQR): 13 (5–20) y Without previous foot ulcer, median (IQR): 8 (3–15) y	Type 1 Type 2 (mainly)	Diabetic patients attending primary care health centres	Duration of diabetes, self‐reported “burning and numbness”, neuropathy symptom severity score, insulin use, previous amputation, goes barefoot outside house (protective), and toenails cut by relative or friend	History of ulcer: 257/2106 (12.2%)	NR	Ulceration	Age, median (IQR): 61 (52–67) y F/M: 175/82 (68%/32%) Diabetes duration, median (IQR): 13 (5–20) y
								NR	92/2106 (4.4%)	NR	Amputation	NR
Jbour, 2003 [60], Jordan	Clinical visits and laboratory analyses	1142	Mean (SD): 56.1 (10.2) y	547 (48%)	Mean (SD): 9 (7.1) y	Type 2	Type 2 diabetic patients from a national referral centre. Exclusion criteria was amputation due to trauma or conditions other than diabetes	NR	Active ulcer: 45 cases (4%)	NR	Ulceration	NR
								Duration of diabetes, smoking, microalbuminuria, retinopathy, hair loss, neurological deficit, absent dorsalis pedis pulses, ulceration, insulin therapy, and HbA1c ≥ 8%.	53 cases (5%)	NR	Amputation	Age: 20–39 y: 3 (5.7%) 40–59 y: 25 (47.2%) ≥ 60 y: 24 (45.3%) F/M: 24/29 (45.3%/54.7%) Diabetes duration: 1–9 y: 22 (41.5%) 10–19 y: 23 (43.4%) ≥ 20 y: 8 (15.1%)
Nyamu, 2003 [18], Kenya	Clinical visits and laboratory analyses	1788	NR	NR	NR	Type 1 Type 2 (mostly)	Patients with type 1 and type 2 diabetes mellitus from a national referral centre	Diabetes duration, neuropathy, poor glycaemic control, diastolic hypertension, dyslipidemia, infection, and poor self‐care	Active ulcer: 82/1788 cases (4.6%)	NR	Ulceration	Age, mean (SD): 56.9 (13.41) y F/M: 41/41 (50%/50%) Diabetes duration, mean (SD): 7.98 (6.86) y
Stein, 2003 [96], Israel	Clinical visits	3995	NR	2090 (52.3%)	NR	Type 1 and type 2	Diabetic outpatients referred to a specialised diabetes clinic	NR	Active ulcer: 49/3995 (1.2%)	NR	Ulceration	NR
Viswanathan, 2005 [107], India	Clinical visits, laboratory analyses, and questionnaires	1319	Mean (SD): 53 (11) y	518 (39.3%)	Mean (SD): 6.9 (5.9) y	Type 2	Patients with type 2 diabetes from four different centres across India	Neuropathy	100/1319 cases (7.6%)	NR	Infection	NR
								NR	30/1319 cases (2.3%)	NR	Amputation	NR
Ndip, 2006 [80], Republic of Cameroon	Medical records, medical interviews, and foot examinations	300	Mean (SD): 56.7 (12.3) y	136 (45.3%)	NR	NR	Diabetic ambulatory and hospitalised patients attending diabetes referral centres (a tertiary‐level facility and a hospital metabolic disease unit). Patients with a documented history of tertiary syphilis and/or leprosy, as well as comatose and aphasic patients, were excluded	Age, diabetes duration, history of foot lesions, neuropathy, and lower extremity deformities	History of ulcer: 37 (12.3%) Active ulcer: 39/300 (13.0%)	NR	Ulceration	NR
								NR	9/300 (3%)	NR	Amputation	NR
Fabian, 2007 [50], Poland	Medical records	355	Mean: 66 y	NR	Mean: 9 y	Type 2	Type 2 diabetes of a family medicine centre	NR	Active ulcer: 15/355 (4.2%)	NR	Diabetic foot	NR
El‐Nahas, 2008 [56], Egypt	Clinical visits	1220	Mean (SD): 50.5 (10.9) y	771 (63.2%)	Mean (SD): 7.9 (5.9) y	Type 1: 61 (5%) Type 2: 1159 (95%)	Diabetic patients attending the diabetes outpatient clinics in a university specialised medical hospital	NR	85/1220 cases (6.9%) History of ulcer: 70 cases (5.7%) Active ulcer: 15 cases (1.2%)	NR	Ulceration	NR
								NR	14/1220 cases (1.1%)	NR	Amputation	NR
Sämann, 2008 [110], Germany	Clinical visits and laboratory analyses	4778	Type 1, mean (SD): 49 (16) y Type 2, mean (SD): 66.4 (10.1) y	2102 (44.0%)	Mean (SD) Type 1: 20.5 (13.5) y Type 2: 7.7 (6.8) y	Type 1: 366 (7.7) Type 2: 4412 (92.3)	Patients with type 1 and type 2 diabetes at the primary care level in Germany. Exclusion criteria: Gestational diabetes, other specific types of diabetes, and not insured with Deutsche BKK	Age, duration of diabetes, height, current smoking, and insulin therapy	138/4778 cases (2.9%) Type 1: 13/366 cases (3.6%; 95% CI 1.9, 6.0) Type 2: 125/4412 cases (2.8%; 95% CI 2.3, 3.4)	NR	Diabetic foot syndrome	Type 1 patients: Age, mean (SD): 61.3 (10.6) y F/M: 6/7 (46%/54%) Diabetes duration, mean (SD): 32.8 (12.4) y Type 2 patients: Age, mean (SD) 70.4 (9.2) y F/M: 38/87 (30%/70%) Diabetes duration, mean (SD): 10.3 (7.7) y
									Type 1: 8 cases (2.2%) 3 acute/5 healed Type 2: 77 cases (1.7%) 37 acute/40 healed	NR	Ulceration/gangrene	NR
									Type 1: 8 cases (2.2%) Type 2: 65 cases (1.5%)	NR	Amputation	NR
Sinharay, 2012 [19], India	NR	1674	NR	NR	0 (analysis conducted at disease onset)	NR	Newly diagnosed diabetes mellitus patients	NR	Active ulcer: 76/1674 (4.54%)	NR	Ulceration	NR
Assaad‐Khalil, 2014 [36], Egypt	Clinical visits	2000	Mean (SD): 57.30 (10.47) Y	1000 (50%)	Mean (SD): 11.76 (8.26) Y	Type 1: Type 2: (96.75%)	Adult (≥ 18‐year‐old) patients with diabetes attending a university diabetic foot screening clinic	Diabetic duration, foot fissures, Charcot's foot, limited joint mobility, peripheral vascular disease, and sensory neuropathy	History of ulcer: 173/2000 cases (8.7%) Active ulcer: 122/2000 cases (6.1%)	NR	Ulceration	Active foot ulceration: F/M: 41/81 (33.6%/66.4%) Past prevalence: 70/103 (40.5%/59.5%)
									88/2000 cases (4.4%)	NR	Amputation	F/M: 26/62 (29.5%/70.5%)
Chiwanga, 2015 [46], Tanzania	Questionnaire, foot examinations	404	Mean (SD): 53.6 (12.7)	224 (55.4%)	< 5: 198 (49.0%) 5–10: 135 (33.4%) > 10: 71 (17.6%)	Type 2	Patients from public diabetic clinics at municipal hospitals	Insulin treatment and peripheral neuropathy	History of ulcer: 107/404 cases Active ulcer: 62/404 cases (15.3%; 95% CI: 2.17–5.92)	NR	Ulceration	Age, mean (SD): 56.4 (13.1) Y F/M: 33/29 (46.8%/53.2%)
Dòria, 2016 [47], Spain	Medical records and patient‐reported anamnesis during routine clinical visits	92	Mean (SD) 70.9 (12.1) y	35 (38.0%)	Mean (SD): 22.3 (12.2) y	Type 2: 81 (88.0%) Other types: 11 (12.0%)	Diabetic patients with chronic kidney disease who were receiving replacement therapy (haemodialysis or peritoneal dialysis) from dialysis centres	Diabetic retinopathy, stenosing carotid plaques, previous stroke	History of ulcer: 18/92 (19.6%) Active ulcer: 16/92 cases (17.4%)	NR	Ulceration	Age, mean (SD): 70.1 (12.6) y F/M: 7/21 (25%/75%) Diabetes duration, mean (SD): 23.4 (11.0) y
									15/92 cases (16.3%)	NR	Amputation	Age, mean (SD): 72.3% (10.3%) y F/M: 5/10 (33.3%/66.7%) Diabetes duration, mean (SD): 23.9 (13.6) y
Sarinnapakorn, 2016 [100], Thailand	Clinical visits and laboratory analyses	593	Mean (SD): 63.80 (13.70) y	387 (65.3)	Mean (SD): 158.00 (104.20) months	Type 2	Adult patients with type 2 diabetes mellitus at a tertiary care hospital	Age, duration of diabetes, eGFR, history of hypertension, dyslipidemia, nephropathy, cardiovascular disease, deformity of foot, numbness, abnormal protective sensation, pulse deficit, history of ulcer and amputation	History of ulcer: 20/593 cases (3.4%)	NR	Ulceration	NR
									13/593 cases (2.2%)	NR	Amputation	NR
Khan, 2017 [78], Pakistan	Clinical visits	230	Mean (SD): 53.82 (9.96) y	136 (59.13%)	Mean (SD): 7.87 (5.50) y	Type 2	Diabetic patients attending diabetes centres	Sex, smoking history, hypertension, coronary artery disease, and stroke	32 (13.91%)	NR	Diabetic foot syndrome	Age, mean (SD): 52.81 (9.27) y Diabetes duration, mean (SD): 8.93 (4.59) y
								NR	History of ulcer: 15/230 (6.52%) Active ulcer: 20 (8.69%)	NR	Ulceration	NR
								NR	3 (1.3%)	NR	Amputation	NR
Mariam, 2017 [72], Ethiopia	Questionnaires, medical records, and patient visits	279	Mean (SD): 49.8 (15.6) y	125 (44.8%)	< 5 y: 155 (55.6%) 6–10 y: 108 (38.7%) 11–15 y: 16 (5.7%)	Type 1: 110 (39.4%) Type 2: 169 (60.6%)	Diabetes mellitus patients who attended the diabetic follow‐up clinic at a university hospital. Those diabetic patients who had any diabetic‐related ulcer were included. Exclusion criteria: Traumatic ulcer due to car accident and diabetic patients who were severely ill and unable to communicate	Residence, types of diabetes mellitus, overweight, obesity, foot self‐care practice, and neuropathy	Active ulcer: 38 cases (13.6%)	NR	Ulceration	F/M: 11/27 (29%/71%) Type 1: 7 cases Type 2: 31 cases
O'Shea, 2017 [86], New Zealand	Interviews and foot examinations	2192	Mean: 63 y	1047 (48%)	Type 1: 147 (13%) y Type 2: 2045 (87%) y	NR	Patients aged over 15 years with either type 1 or type 2 diabetes registered with the Waikato Regional diabetes service mobile retinal photo screening service. Exclusion criteria: Patients with established retinopathy, who attend a specialist eye clinic, as they are now monitored by an ophthalmologist	Age, type and duration of diabetes, and smoking	History of ulcer: 110/2192 cases (5.0%) Active ulcer: 2/2192 (0.1%)	NR	Ulceration	NR
									22/2192 cases (1.0%)	NR	Amputation	NR
Galstyan, 2018 [52], Russia	National disease registry	NR	> 18 y	NR	NR	NR	General population (81 regions of the Russian Federation)	NR	Type 1: 506.3 (2013) to 473.6 (2016) per 10,000 patients (4.7%) Type 2: 214.6 (2013) to 194.8 (2016) per 10,000 patients (1.9%)	NR	Diabetic foot	Age Type 1: 44.9 (2013) to 46.8 (2016) y Type 2: 64.4 (2013) to 66.2 (2016) y Diabetes duration Type 1, mean: 15.4 (2013) to 19.0 (2016) y Type 2, mean: 7.4 (2013) to 10.1 (2016) y
								NR	Type 1: 10.5 (2013) to 12.4 (2016) per 10,000 patients Type 2: 9.6 (2013) to 10.9 (2016) per 10,000 patients	NR	Amputation	Age Type 1: 51.4 (2013) to 51.7 (2016) y Type 2: 65.9 (2013) to 66.2 (2016) y Diabetes duration Type 1: 18.4 (2013) to 21.3 (2016) y Type 2: 9.1 (2013) to 9.9 (2016) y
Tindong, 2018 [98], Republic of Cameroon	Questionnaires and clinical visits	203	26–96 y	128 (63.1%)	Median (IQR): 4.0 (1 0.0–8.0) y	Type 2: 198 (97.5%) Type 1: 5 (2.5%)	Diabetic outpatients (diabetes clinics) and inpatients (medical and surgical wards), older than 21 years. Exclusion criteria: Acute states	Peripheral arterial disease and loss of protective sensation	History of ulcer: 28/203 (13.8%) Active ulcer: 24/203 (11.8%)	NR	Ulceration	Age, mean (SD): 55.4 (10.8) y F/M: 13/11 (54.2%/45.8%) Diabetes duration, median (IQR): 2.5 (1.0–6.0) y
								NR	2/203 (1.0%)	NR	Amputation	NR
Vibha, 2018 [106], India	Questionnaires and clinical visits	620	Mean (SD): 63.37 (10.8) y	356 (57.4%)	Median (IQR): 7 (3–13) y	Type 2	Adult diabetic patients from rural areas of Udupi district. Exclusion criteria: Gestational diabetes mellitus, stroke, bilateral below‐knee amputation, Hansen's disease, and foot deformities secondary to other causes	Age, low socioeconomic status, sedentary physical activity, and duration of diabetes	321/620 cases (51.8%)	NR	Diabetic foot syndrome	Age < 50 y: 18 (5.6%) 51–60 y: 62 (19.3%) 61–70 y: 130 (40.5%) > 70 y: 111 (34.6%) F/M: 173/148 (53.9%/46.1%) Diabetes duration 0–5 y: 95 (29.5) 6–10 y: 100 (31.2) > 10 y: 126 (39.3)
								NR	History of ulcer: 61/620 (9.8%)	NR	Ulceration	NR
								NR	9/620 (1.5%)	NR	Amputation	NR
Yazdanpanah, 2018 [113], Iran	Patient interviews and clinical examinations	605	Mean (SD): 53.82 (10.73) y	346 (57.2%)	Mean (SD): 9.18 (7.06) y	Type 1: 14 (2.3%) Type 2: 591 (97.7%)	Diabetic patients, over 18 years old, who attended a university hospital diabetes clinic with or without diabetic foot ulcers. Exclusion criteria: Disabling diseases or psychological disorders	Diabetes duration > 20y, BMI > 25, 10g monofilament sensation, abnormal ankle brachial Index, and low education	History of ulcer: 37/605 cases Active ulcer: 39/605 cases (6.4%; 95% CI 4.64–8.73)	NR	Ulceration	Age < 40 y: 1 (1.6%) 40–49 y: 7 (5.6%) 50–59 y: 18 (7.9%) ≥ 60 y: 13 (6.8%) F/M: 17/22 (43.6%/56.4%) Diabetes duration ≤ 5: 6 (2.6%) 6–10: 10 (5.7%) 11–20: 18 (11.2%) > 20: 5 (13.2%)
								NR	6/605 cases	NR	Amputation	NR
Younis, 2018 [114], Pakistan	Medical records, clinical visits, and laboratory analyses	1940	Mean (SD): 51.24 (10.60) y	1222 (63%)	Mean (SD): 7.29 (6.1) y	Type 2	Patients ≥ 30 years with type 2 diabetes presenting to a specialist diabetic clinic. Cognitive impairment and hypothyroidism were exclusion criteria	Age, duration of diabetes, HbA1C, peripheral neuropathy, and peripheral arterial disease	Active ulcer: 136/1940 cases (7.02%)	NR	Ulceration	Age, mean (SD): 53.73 (9.98) y Diabetes duration, mean (SD): 9.96 (6.35) y
Lin, 2019 [68], Taiwan	Insurance claims data	From 1,326,422 (2005) to 2,200,626 (2014)	NR	NR	NR	Type 1 and type 2	Patients (< 100 y) with diabetes who had visited outpatient clinics at least three times or been hospitalised at least once within 365 calendar days	NR	2005: 17,756/1,326,422 (1.34%) 2014: 23,179/2,200,626 (1.05%)	NR	Diabetic foot (infection and/or amputation)	NR
								NR	2005: 16,913/1,326,422 (1.28%) 2014: 22,008/2,200,626 (1.00%)	NR	Infection	Age < 40 y: 612 (2.8%) 40–65 y: 8483 (38.5%) > 65 y: 12,913 (58.7%) F/M: 9626/12,382 (43.7%/56.3%)
								NR	2005: 4295/1,326,422 (0.32%) 2014: 4049/2,200,626 (0.18%)	NR	Amputation	NR
Lee, 2020 [64], Australia	Routine clinical visits and laboratory analyses	2120	Mean (SD): 44.3 (15.6) y	970 (45.8%)	Mean (SD): 18.5 (12.5) y	Type 1	Adult (> 18 y) patients with type 1 diabetes attending specialist centres	Metabolic syndrome	Active ulcer: 96/1958 (4.90%)	NR	Ulceration	NR
								Metabolic syndrome	32/1619 (1.98%)	NR	Amputation	NR
Abdelbagi, 2021 [32], Sudan	Questionnaire and clinical examination	2048	Median (IQR): 58 (15) y	1100 (54.2%)	Median (IQR): 5 (6) y	Type 1: 613 (29.9%) Type 2: 1435 (70.1%)	Patients aged 18 years and above with type 1 or type 2 diabetes. Exclusion criteria: Patients with traumatic wounds or malignancy‐related ulcers	Age, duration of diabetes, type 2 diabetes, family history of diabetes, hypertension, and obesity.	Active ulcer: 260/2048 (12.7%)	NR	Diabetic foot	Age, median (IQR): 69 (4) y F/M: 127/133 (48.8%/51.2%) Type 1: 67 (25.8%) Type 2: 193 (74.2%) Diabetes duration, median (IQR): 6 (4) y
Galvão, 2021 [53], Brasil	Medical records, interviews	229	With ulcer, mean (SD): 62 (12.8) y Without ulcer, mean (SD): 63.5 (14.2) y	91 (39.7%)	NR	Type 1 and type 2	All adult hospitalised patients with type 1 or type 2 diabetes	Peripheral artery disease. Emollient and anticoagulant use were protective	Active ulcer: 60/229 (26.2%)	NR	Ulceration	Age, mean (SD): 62 (12.8) y F/M: 21/39 (35%/75%)
Tola, 2021 [99], Ethiopia	Medical records from governmental hospitals (including follow‐up clinics data)	502	Mean (SD): 48.13 (14.77) y	215 (42.8%)	Median (IQR): 28 months (14–40.25) y	Type 2	Type 2 diabetes mellitus patients attending chronic follow‐up clinics at governmental hospitals. Type 2 patients with unidentified diabetic foot status were excluded	Hypertension, marital status (unmarried), physical inactivity, insulin use, obesity, delay in initiation of follow‐up, and infection history	NR	Active ulcer: 106/502 (21.1%) during 5‐y period	Ulceration	Age (years): 15–29: 6 (12.2%) 30–44: 23 (14.7%) 45–59: 29 (19.2%) 60 and older: 48 (32.9%)
Akhtar, 2022 [34], Pakistan	Questionnaires	1503	Mean (SD): 51.58 (11.49) y	999 (66.5%)	Mean (SD): 5.652 (3.812) y	Type 2: 79% Others: 21%	Adult diabetic patients from the general population	Age, overweight, obesity, and duration of diabetes	Active ulcer: 253/1503 (16.83%; 95% CI: 14.9–18.7%)	NR	Ulceration	Age 18–34 y: 1 (0.40%) 35–54 y: 89 (35.18%) 55–74 y: 137 (54.14%) 75+ y: 26 (10.28%) F/M: 175/78 (69.17%/30.83%)
Bundó, 2022 [40], Spain	Primary health care database	394,266	Mean (SD): 70.3 (12.5) y	177,558 (45%)	Mean (SD): 10.2 (6.57) y	Type 2	All live adult subjects (age > 18 y) with type 2 in the primary care database	Increasing age, male sex, diabetes duration, at‐risk alcohol use, higher BMI, hypertension, macrovascular and microvascular complications, diabetic neuropathy, peripheral artery disease, insulin use, and previous history of diabetic foot disease. Non‐insulin antidiabetic drugs, hyperlipidaemia, lifestyle, and dietary measures were negatively associated with diabetic foot disease.	Diabetic foot: 3277/394,266 (0.83%) History of ulcer: 10,852/394,266 (2.75%)	NR	Diabetic foot	Age, mean (SD): 74.0 (12.0) y F/M: 1187/2090 (36.2%/63.8%) Diabetes duration, mean (SD): 13.4 (7.35) y
								NR	Active ulcer: 2687/394,266 (0.682%)	NR	Ulceration	NR
								NR	943/394,266 (0.24%)	NR	Amputation	NR
El Alami, 2022 [49], Morocco	Survey (face‐to‐face interviews)	505	Mean (SD): 57.27 (10.74) y	430 (85.15%)	< 5 y: 148 (29.4%) 5–10 y: 165 (32.8%) > 10 y: 190 (37.8%)	Type 2	Patients with type 2 diabetes aged 20 y or older and treated in health centres	NR	Active ulcer: 14/505 (2.8%)	NR	Diabetic foot	F/M: 11/3 (78.6%/21.4%)
Negash, 2022 [81], Ethiopia	Questionnaires (face‐to‐face interviews), clinical visits, and laboratory analyses	267	Mean (SD): 49.9 (15.7) y	119 (44.6%)	< 5 y: 136 (50.9%) 5–10 y: 53 (19.9%) > 10 y: 78 (29.2%)	Type 1: 69 (25.8%) Type 2: 198 (74.2%)	Adult patients (18 y old and older) with diabetes mellitus who attended an ambulatory clinic of a referral hospital. Exclusion criteria: Traumatic ulcers resulting from road traffic accidents and severe illness and inability to communicate	Area of residence, lack of regular exercise, peripheral neuropathy, and foot calluses	Active ulcer: 30/267 (11.2%; 95% CI: 7.42–15.05)	NR	Ulceration	Age, years: < 30 y: 7 (23.3%) 30–39 y: 8 (26.7%) 40–49 y: 3 (10.0%) ≥ 50 y: 12 (40.0%) Diabetes duration < 5 y: 9 (30.0%) 5–10 y: 6 (20.0%) ≥ 10 y: 15 (50.0%)
Ponirakis, 2022 [91], Qatar, Kuwait, and Kingdom of Saudi Arabia	Clinical visits and laboratory analyses	3021	Mean (SD): 57.9 (11.7) y	1408/3011 (46.8)	Mean (SD): 14.4 (9.2) y	Type 2	Patients (aged 18–85 years) with type 2 diabetes in secondary healthcare diabetes centres. Chemotherapy, severe vitamin B12 deficiency, and hypothyroidism were exclusion criteria	Diabetic peripheral neuropathy, hypertension, and moderate vitamin D deficiency	Active ulcer: 88/3021 (2.9%)	NR	Ulceration	NR
UroojNajami, 2022 [104], India	Medical records, interviews, and clinical visits	500	Mean (SD): 55 (15) y	260 (52%)	Mean (SD): 8.9 (6.5) y	Type 1: 55 (11%) Type 2: 445 (89%)	Diabetic patients attending the outpatients department of general surgery in a private medical college	Gender, type of DM, neuropathy, duration of DM, HbA1c	Active ulcer: 24/500 cases (4.8%)	NR	Ulceration	Data inconsistency
								NR	140/500 (28%)	NR	Infection	NR
								NR	10/500 cases (2%)	NR	Amputation	NR
Hirpa, 2023 [57], Ethiopia	Questionnaires (face‐to‐face interviews) medical charts, clinical visits, and laboratory analyses	278	Mean (SD): 46.07 (15.6) y	141 (50.7%)	< 5 y: 162 (58.3) 5–10 y: 72 (25.9) ≥ 10 y: 44 (15.8)	Type 1: 79 (28.4%) Type 2: 199 (71.6%)	Diabetes patients (≥ 18 y) attending the follow‐up clinic for chronic illness of a public hospital	Marital status, diabetes peripheral neuropathy, and deformity	Active ulcer: 52/278 (18.7%; 95%; CI 14.1, 23.3)	NR	Ulceration	Age: < 30 y: 8 (15.4%) 30–50 y: 21 (40.4%) 50+ y: 23 (44.2%)
Kilic, 2024 [66], Turkey	Face‐to‐face interviews, foot examinations	357	NR	NR	With ulcer, mean (SD): 14.21 (7.42) y Without ulcer, mean (SD): 9.96 (6.91) y	Type 1: 30 (8.4%) Type 2: 327 (91.6%)	Adult patients with diabetes mellitus who presented to the diabetes and internal medicine outpatient clinics of the hospitals. Exclusion criteria: Gestational diabetes mellitus, stroke, and bilateral below‐knee amputation	Peripheral arterial disease, history of ulcer, oedema, fungus between the toes	History of ulcer: 53/357 (14.8%) Active ulcer: 61/357 cases (17.1%; 95% CI 13.2–21.5)	NR	Ulceration	Age, mean (SD): 60.22 (13.15) y F/M: 32/29 (52.5%/47.5%) Type 1: 5 (8.2%) Type 2: 56 (91.8%) Diabetes duration, mean (SD): 14.21 (7.42) y
								NR	8/357 (2.2%)	NR	Amputation	NR
Kuguyo, 2024 [61], Zimbabwe	Face‐to‐face interviews, medical records, and foot examinations	352	Mean (SD): 57.9 (14) y	270 (76.7%)	< 1 y: 71 (20.2%) 1.1–5 y: 118 (33.5%) 6–10 y: 58 (16.5%) 11–15 y: 54 (15.3%) > 15 y: 51 (14.5%)	Type 1: 62 (17.6%) Type 2: 290 (82.4%)	Patients from outpatient diabetes treatment facilities (14 primary care clinics and 2 referral hospitals). Exclusion criteria: Pregnant women and individuals admitted for inpatient care	NR	187/352: 53% (95% CI 50–56)	NR	Diabetic foot	NR
								Peripheral neuropathy (burning, aching pain or tenderness of feet)	History of ulcer or active ulcer: 59/352 cases (17%) Active ulcer: 10/352 (2.84%)	NR	Ulceration	Diabetes duration (past or active/active) ≤ 5 y: 14.4%/3.2% 6–10 y: 11.3%/3.8% 11–15 y: 30.0%/0.0% > 15 y: 27.5%/3.9%
								Not insulin use	7/352 cases (3%)	NR	Amputation	NR

Abbreviations: BMI, Body Mass Index; GP, General practitioner; NR, Not reported or not applicable or not assessed; SD, Standard deviation.

^a^ Risk factors associated with the development of diabetic foot complications were identified by the study authors through statistical analyses.

^b^ Cumulative incidence; mean (SD) follow‐up, years: P4P group, 4.6 (3.0); non‐P4P group, 3.9 (2.8).

Twenty‐six of the included studies [35, 40, 42, 44, 45, 48, 51, 55, 62, 67, 68, 70, 71, 73, 76, 85, 90, 92, 93, 95, 97, 101, 105, 109, 111, 115] evaluated the epidemiology of DF disease searching for DF‐related diagnostic and procedural codes in real‐world data sources (e.g., claims databases, electronic health records, diabetes registries) (Table 2 shows the different diagnostic and procedural coding systems and codes used to define DF disease), whereas the others relied mainly on primary data, such as standardized physical, laboratory, and instrumental examinations, or other types of secondary data (e.g., paper‐based health records).

**TABLE 2:** Data sources and diagnostic/procedural codes used to define diabetic foot complications in the included studies.

Author, year	Diabetic foot definition	Data source	Classification system used	Codes
Prospective cohort studies				
Rith‐Najarian, 1993	Amputation	Clinic amputation registry	ICD‐9 (Procedures)	84.10–84.17
Monami, 2009	Ulceration	Hospital discharge records	ICD‐9‐CM	707 or 440.23
Williams, 2010	Ulceration	Administrative databases	ICD‐9	707.1x, 707.8–707.9, 681.1x, 681.9x, 682.7x, 730.06–730.09, 730.17, 730.19, 730.27, 730.29, 785.4x
Robinson, 2015	Amputation	Electronic hospital records	ICD9‐CM‐A	V4970, V4971, V4972, V4973, V4974, V4975, V4976, V4977, V4978
			ICD‐10‐AM	4433800, 4435800, 4436100, 4436101, 4436400, 4436401, 4436700, 4436701, 4436702, 4437000, 4437600, 9055700
Park, 2016	Diabetic foot	Insurance claims records	KCD‐6	L97, R02, Z89.4, Z89.5, Z89.6, Z89.7, Z89.8, Z89.9, S80.7, S80.8, S80.9, S81.0, S81.7, S81.8, S81.9, S90.7, S90.8, S90.9, S91.0, S91.1, S91.2, S91.3, S91.7, T00.3, T04.3, T13.0, T13.1, T13.3, T13.4, T13.5, T13.8, T13.9
Retrospective cohort studies				
Most, 1983	Amputation	Discharge data from hospital abstracting services	ICDA‐8 (Procedures)	85.5–85.9
			HICDA‐2	84.5–84.9
Waugh, 1988	Amputation	Hospital discharge records	ICD‐9	871–877, V52, V53.8, V54.8, V54.9
Ebskov, 1991	Amputation	Hospital discharge records	ICD	NR
Deerochanawong, 1992	Amputation	Surgery operation records, hospital discharge records	OPCS‐4	X09‐X11
Farrell, 1993	Amputation	Clinical registry	ICD‐9	84.11, 84.13–84.19
Valway, 1993	Amputation	Hospital discharge records	ICD‐9	84.10–84.19
New, 1998	Amputation	Diabetes registry	OPCS‐4	X07‐X11
Lavery, 1999	Amputation	Medical records	ICD‐9‐CM	84.11–84.18, 84.30
Ramsey, 1999	Ulceration	Administrative databases	ICD‐9‐CM	041, 681.1, 682.6, 682.7, 682.9, 707.1
	Infection (osteomyelitis)		ICD‐9‐CM	730
	Amputation		CPT‐4	28810, 28820
			ICD‐9‐CM	84.10–84.15, 84.17
Schofield, 2009	Amputation	Hospital discharge database	OPCS‐4	X09‐X12
Jørgensen, 2013	Amputation	Administrative patient registry	ICD‐10	Z89.4, Z89.5–Z89.7
Anderson, 2017	Ulceration	Primary care electronic health records	READ	NR
Heald, 2019	Ulceration	Electronic health records	READ	NR
Chou, 2020	Infection	Population‐based health insurance database	ICD‐9‐CM	040.0x, 250.7x, 440.2x, 680.7x, 681.1x, 682.7x, 707.1x, 730.07, 730.17, 730.27, 730.97, 785.4x
	Amputation		ICD‐9‐CM	V49.71, V49.72, V49.73, V49.74, V49.75, V49.76, V49.77, V52.1x
			ICD‐9‐CM (Procedures)	84.11–84.17
Déruaz‐Luyet, 2020	Ulceration	Commercial insurance claims database	ICD‐9‐CM	440.23, 707.1, 707.14, 707.15
	Infection (cellulitis, osteomyelitis, gangrene)		ICD‐9‐CM	040.0, 440.24, 440.2 + 785.4, 250.7 + 785.4, 680.7, 681.1, 681.10, 682.7, 730.07, 730.17, 730.27, 730.97
	Amputation		ICD‐9‐CM	84.10, 84.11, 84.12, 84.13, 84.14, 84.15, 84.16, 84.17, 84.18, 84.19
			CPT‐4	27290, 27295, 27590–27598, 27880–27889, 28800, 28805, 28810, 28820, 28825
Li, 2020	Ulceration	Hospital medical records	ICD‐9‐CM	440.23, 707.1, 707.14, 707.15
	Amputation		ICD‐9‐CM (Procedures)	84.10, 84.11, 84.12, 84.13, 84.14, 84.15, 841.16, 84.17, 84.18, 84.19
Lu, 2021	Ulceration	Population‐based health insurance database	ICD‐9‐CM	440.23, 707.1x
	Infection (gangrene)		ICD‐9‐CM	440.24, 785.4
Tai, 2021	Ulceration	Population‐based health insurance database	ICD‐9‐CM	707.06, 707.07, 707.1, 892, 893
Lovell, 2022	Ulceration	Electronic medical records	ICD	NR
Cross‐sectional studies				
Lin, 2019	Diabetic foot	Insurance claims data	ICD‐9	(040.0x, 785,4x, or 440.24) with (250.7x, 440.2x, 440.21, 440.22, or 440.23) 440.23, 680.7, 681.1, 681.10, or 682.7, 707.1x, 707.14, 707.15, 730.07, 730.17, 730.27, 730.97, V49.71‐V49.77, V52.1x, 84.11–84.17
	Infection (gangrene, osteomyelitis, and cellulitis or abscess)		ICD‐9	(040.0x, 785,4x, or 440.24) with (250.7x, 440.2x, 440.21, 440.22, or 440.23) 440.23, 680.7, 681.1, 681.10, or 682.7, 707.1x, 707.14, 707.15, 730.07, 730.17, 730.27, 730.97
	Amputation		ICD‐9	V49.71‐V49.77, V52.1x, 84.11–84.17
Bundó, 2022	Diabetic foot	Primary health care database	ICD‐10	L97, E11.621, M86, I96, E11.52, Z89, M14.6, E11.61
			ICD‐10 (Procedures)	0Y6
	Ulceration		ICD‐10	L97, E11.621
	Infection (osteomyelitis, gangrene)		ICD‐10	M86, I96, E11.52
	Amputation		ICD‐10	Z89
			ICD‐10 (Procedures)	0Y6

Abbreviations: CPT‐4, Current Procedural Terminology—Version 4; HICDA‐2, Hospital International Classification of Disease Adaptation—version 2; ICD, International Classification of Diseases; ICD‐10, ICD ‐ 10th revision; ICD‐10‐AM, ICD‐10—Australian Modification; ICD‐9, ICD—9th revision—Clinical Modification; ICD‐9‐CM, ICD‐9—Clinical Modification; ICD‐9‐CM‐A, ICD‐9‐CM—Australian; ICDA‐8, ICD Adapted—8th revision; KCD‐6, Korean Standard Classification of Diseases—6th version; OPCS‐4, Office of Population Censuses and Surveys Classification of Surgical Operations and Procedures—4th revision.

### 3.3 Incidence Rates of Diabetic Foot Complications

#### 3.3.1 Incidence of Ulcerations

**FIGURE 2:**
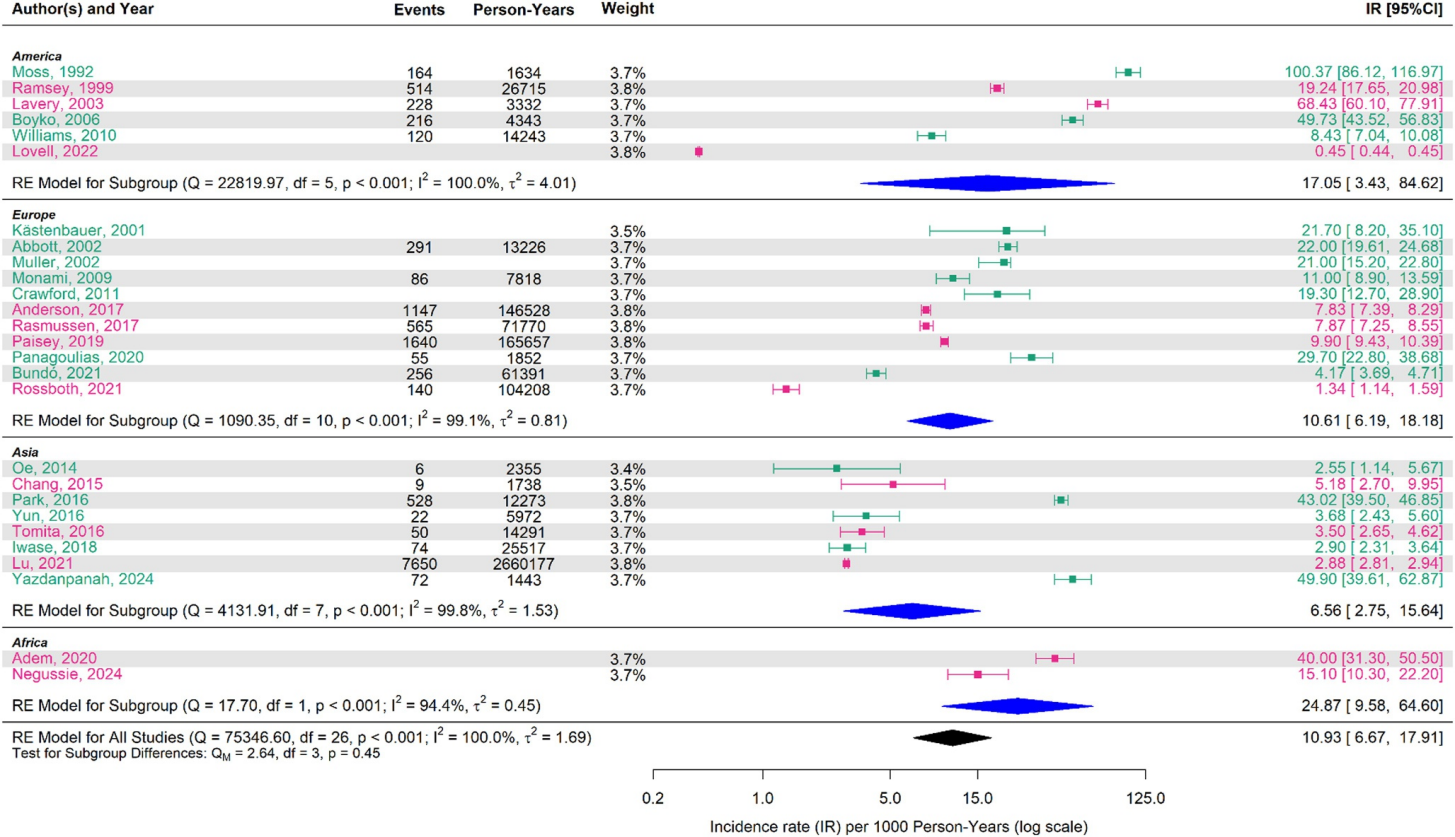
Forest plot showing the estimated incidence of ulcerations. Studies are stratified by geographical zone and coloured according to their design, with green and magenta used in prospective and retrospective studies, respectively. The summary polygon below each subgroup (filled in blue) shows the results of a fixed or random‐effects model for just the studies within that group. The summary polygon at the bottom of the plot shows the results of the model when all studies are analysed. The test for subgroup difference (Q_M_) reflects the statistical significance of the subgroup covariate when included in a meta‐regression model. The number of events and person‐years is reported only for studies with available information. I^2^, Inconsistency measure; Q, Cochran's Q statistic, along with degrees of freedom (df) and its *p*‐value; RE, Random‐effects; *τ* ^2^, Between‐study variance.

Twenty‐seven cohort studies contributed to the analysis (Figure 2). The random‐effects pooled incidence rate of DF ulcerations was 10.93 (95% CI, 6.67–17.91) cases per 1000 person‐years, with marked between‐study heterogeneity (*Q* = 75,346.6, df = 26, *p* < 0.001; I^2^ = 100%; *τ* ^2^ = 1.69). When stratified by geographical region, the pooled subgroup incidence rates were 17.05 (95% CI, 3.43–84.62) cases per 1000 person‐years in America, 10.61 (95% CI, 6.19–18.18) in Europe, 6.56 (95% CI, 2.75–15.64) in Asia, and 24.87 (95% CI, 9.58–64.60) in Africa. No studies from Oceania could be included in this meta‐analysis.

Visual inspection of the funnel plot suggested no marked asymmetry, with studies clustering in the upper part due to their high precision. Nevertheless, Begg and Mazumdar's rank correlation test identified a significant negative correlation between effect size and study precision (Kendall's *τ* = −0.54, *p* < 0.001) (Supporting Information S1: Figure S1). This result may be influenced by the presence of two highly precise studies [70, 71] rather than the presence of publication bias. To further investigate this finding, the Trim and Fill method was applied and identified only one potentially missing study (Supporting Information S1: Figure S2). Incorporating this imputed study did not materially alter the results, and the rank correlation remained significant (Kendall's *τ* = −0.49, *p* < 0.001). When the two studies with the highest precision were excluded, Begg and Mazumdar's test was no longer significant (Kendall's *τ* = −0.02, *p* = 0.91), indicating that the initial finding was largely driven by these two highly weighted studies (Supporting Information S1: Figure S3). Overall, these results suggest that no substantial asymmetry was present, and the negative correlation observed with Kendall's *τ* was primarily attributable to the influence of a small number of highly precise studies, rather than providing definitive evidence of publication bias.

In the meta‐regression analysis (Table 3), the publication year and the study period accounted for the largest reductions in between‐study heterogeneity (*R* ^2^ = 23.9% and 18.7%, respectively). Of these two covariates, only publication year showed a statistically significant non‐zero regression coefficient (Wald test *p* = 0.003); however, its inclusion in the model did not lead to a meaningful change in the pooled estimate. In contrast, the inclusion of the study period led to a slight shift in the pooled estimate (9.56 cases per 1000 person‐years; 95% CI, 6.01–15.20).

**TABLE 3:** Results from meta‐regression for both the incidence of ulcerations and amputations, along with an assessment of residual heterogeneity.

Outcome	Covariate	Meta‐regression analysis				Heterogeneity assessment	
		*p* ^e^	Cochran's Q (df)	*p* (*Q* test)	Pooled estimate	*τ* ^2^ ^f^	*R* ^2^ (%)^g^
Ulcerations	None (random‐effects MA)	—	75346.60 (df = 26)	*p* < 0.001	10.93 [6.67, 17.91]	1.69	—
	Study's publication year	*p =* 0.003	37071.66 (df = 25)	*p* < 0.001	10.95 [7.11, 16.86]	1.29	23.9
	Age at enrolment (years)	*p =* 0.300	36513.14 (df = 24)	*p* < 0.001	11.07 [6.64, 18.46]	1.75	0.0
	Sex (% of women)	*p =* 0.270	12138.50 (df = 24)	*p* < 0.001	10.66 [6.41, 17.71]	1.72	0.0
	Diabetes duration (years)	*p =* 0.890	4904.75 (df = 17)	*p* < 0.001	13.47 [7.69, 23.60]	1.53	9.7
	Follow‐up (years)	*p =* 0.370	40585.42 (df = 21)	*p* < 0.001	10.66 [5.99, 18.98]	1.97	0.0
	Study period (years)^d^	*p =* 0.070	11554.98 (df = 22)	*p* < 0.001	9.56 [6.01, 15.20]	1.37	18.7
	Study design (retrospective vs. prospective study)	*p =* 0.110	55114.56 (df = 25)	*p* < 0.001	10.95 [6.79, 17.67]	1.59	6.1
	Diabetic foot identification method (diagnostic codes vs. others)	*p* = 0.280	47339.29 (df = 25)	*p* < 0.001	10.95 [6.70, 17.91]	1.68	0.8
	Data source^b^	*p* = 0.180	25384.61 (df = 23)	*p* < 0.001	10.95 [6.81, 17.62]	1.57	7.3
	Setting^c^	*p =* 0.830	34472.31 (df = 23)	*p* < 0.001	10.93 [6.54, 18.29]	1.84	0.0
	Geographical zone^a^	*p =* 0.450	28059.93 (df = 23)	*p* < 0.001	10.90 [6.63, 17.93]	1.72	0.0
Amputations	None (random‐effects MA)	—	8500.51 (df = 23)	*p* < 0.001	3.58 [1.93, 6.63]	2.31	—
	Study's publication year	*p =* 0.056	8386.29 (df = 22)	*p* < 0.001	3.56 [1.98, 6.39]	2.08	9.9
	Age at enrolment (years)	*p =* 0.540	7641.41 (df = 19)	*p* < 0.001	3.59 [1.77, 7.28]	2.66	0.0
	Sex (% of women)	*p =* 0.610	3492.89 (df = 16)	*p* < 0.001	3.64 [1.75, 7.58]	2.43	0.0
	Diabetes duration (years)	*p =* 0.520	2312.35 (df = 14)	*p* < 0.001	2.82 [1.42, 5.59]	1.87	19.1
	Follow‐up (years)	*p =* 0.032	3144.96 (df = 13)	*p* < 0.001	2.19 [1.09, 4.43]	1.83	20.6
	Study period (years)^d^	*p =* 0.090	7179.65 (df = 20)	*p* < 0.001	3.48 [1.90, 6.38]	2.13	7.7
	Study design (retrospective vs. prospective study)	*p =* 0.560	8313.58 (df = 22)	*p* < 0.001	3.58 [1.92, 6.70]	2.38	0.0
	Diabetic foot identification method (diagnostic codes vs. others)	*p* = 0.170	6685.01 (df = 22)	*p* < 0.001	3.55 [1.93, 6.51]	2.38	0.0
	Data source^b^	*p* = 0.800	7936.02 (df = 20)	*p* < 0.001	3.58 [1.88, 6.82]	2.53	0.0
	Setting^c^	*p =* 0.770	7456.51 (df = 20)	*p* < 0.001	3.54 [1.85, 6.75]	2.53	0.0
	Geographical zone^a^	*p =* 0.024	8051.36 (df = 20)	*p* < 0.001	3.51 [2.03, 6.08]	1.82	21.3

Abbreviations: df, Degrees of freedom referred to the Cochran's Q test; MA, Meta‐analysis.

^a^ The following geographical regions were considered: America, Europe, Asia, Africa, and Oceania.

^b^ The following types of data sources were considered: primary data collection only, multiple data sources, healthcare data sources, and other databases.

^c^ The following settings were considered: population‐/registry‐based, primary/community care‐based, specialist outpatient clinic‐based, and hospital‐based/inpatient or tertiary care.

^d^ The study period is defined by the combination of two study‐level covariates: the calendar year in which the study starts and the study duration, calculated as the difference between the study end year and the study start year. Both covariates were included in the model.

^e^ *p*‐value from omnibus Wald‐type test of parameters (i.e., study‐level covariates included in the model).

^f^ Between‐study variance was estimated before (total) and after (residual) inclusion of each study‐level covariate in the model.

^g^ *R* ^2^ is the proportion of the total between‐study variance that is ‘explained’ (i.e., reduced) by the effect of the study‐level covariate included in the model. Negative values are set to zero.

#### 3.3.2 Incidence of Amputations

**FIGURE 3:**
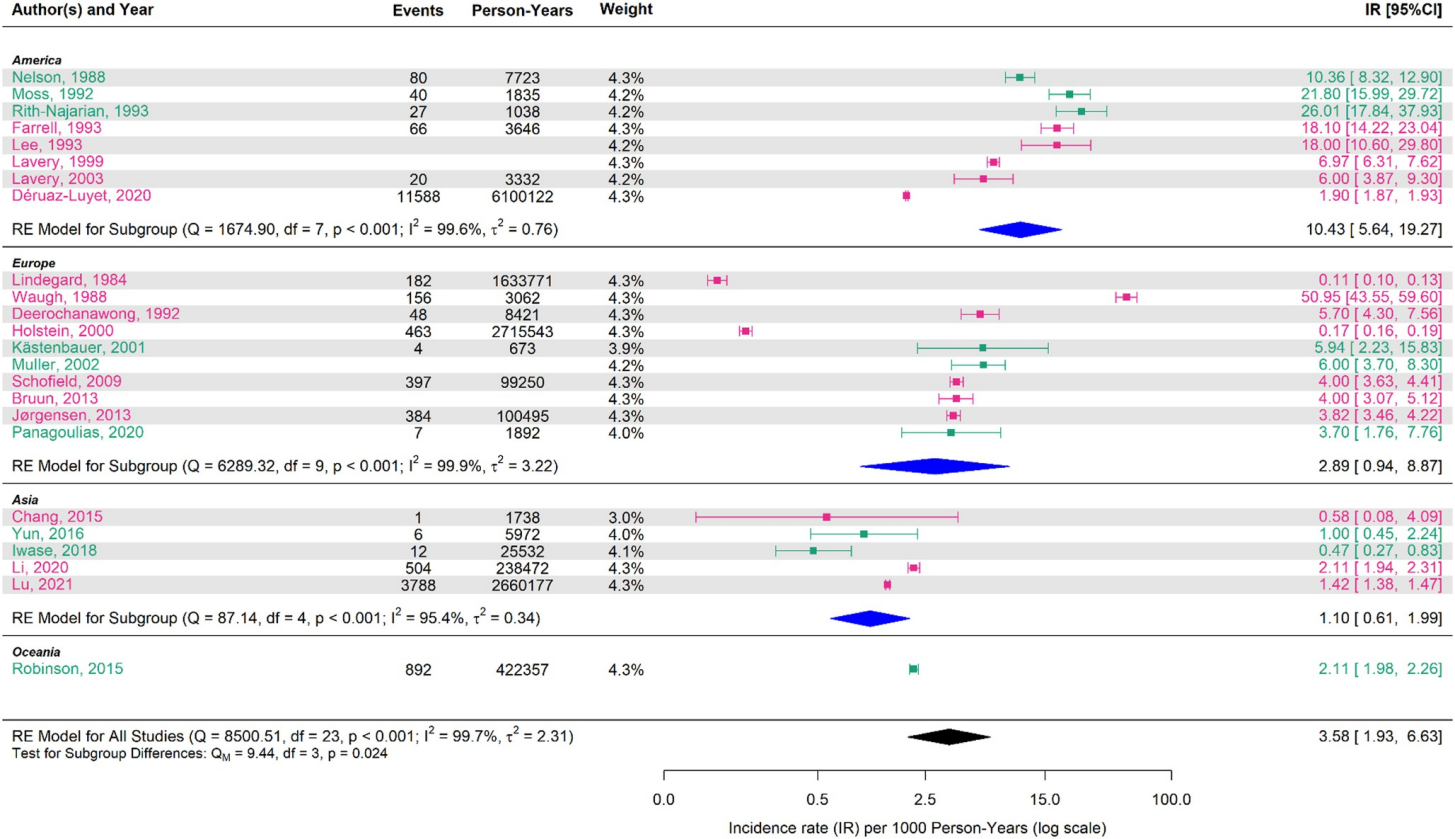
Forest plot showing the estimated incidence of amputations. Studies are stratified by geographical zone and coloured according to their design, with green and magenta used in prospective and retrospective studies, respectively. The summary polygon below each subgroup (filled in blue) shows the results of a fixed or random‐effects model for just the studies within that group. The summary polygon at the bottom of the plot shows the results of the model when all studies are analysed. The test for subgroup difference (Q_M_) reflects the statistical significance of the subgroup covariate when included in a meta‐regression model. The number of events and person‐years is reported only for studies with available information. I^2^, Inconsistency measure; Q, Cochran's Q statistic, along with degrees of freedom (df) and its *p*‐value; RE, Random‐effects; *τ* ^2^, Between‐study variance.

Across 24 cohort studies, the random‐effects pooled incidence of lower‐limb amputations was 3.58 (95% CI, 1.93–6.63) cases per 1000 person‐years (Figure 3). Heterogeneity was again substantial (*Q* = 8500.5, df = 23, *p* < 0.001; I^2^ = 99.7%; *τ* ^2^ = 2.31). When stratified by geographical region, the pooled subgroup incidence rates were 10.43 (95% CI, 5.64–19.27) cases per 1000 person‐years in America, 2.89 (95% CI, 0.94–8.87) in Europe, and 1.10 (95% CI, 0.61–1.99) in Asia. As for Oceania, only one study was included in the meta‐analysis, reporting an incidence rate of 2.11 (95% CI, 1.98–2.26) cases per 1000 person‐years.

Visual inspection of the funnel plot revealed no marked asymmetry, and Begg and Mazumdar's rank correlation test showed no significant correlation between effect size and study precision (Kendall's *τ* = −0.11, *p* = 0.48) (Supporting Information S1: Figure S4). In the meta‐regression analysis (Table 3), geographical zone, length of follow‐up, and diabetes duration accounted for the largest reductions in between‐study heterogeneity (*R* ^2^ = 21.3%, 20.6% and 19.1%, respectively). Among these covariates, only geographical zone and follow‐up duration showed statistically significant non‐zero regression coefficients (Wald tests *p* = 0.024 and *p* = 0.032, respectively). Furthermore, the inclusion of follow‐up duration and diabetes duration in the model led to substantial shifts in the pooled estimate, yielding 2.19 (95% CI, 1.09–4.43) and 2.82 (95% CI, 1.42–5.59) cases per 1000 person‐years, respectively.

#### 3.3.3 Incidence of Infections

The forest plot in Supporting Information S1: Figure S5 summarises infection rates. Only 2 studies were included, yielding a random‐effects pooled incidence of DF infections of 19.11 (95% CI, 4.81–75.97) cases per 1000 person‐years. Heterogeneity was substantial (*Q* = 11.42, df = 1, *p* < 0.001; I^2^ = 91.2%; *τ* ^2^ = 0.91), largely reflecting the opposing incidence estimates reported by the two studies, which limits the interpretability of the pooled estimate.

### 3.4 Prevalence of Diabetic Foot Complications

#### 3.4.1 Prevalence of Active Ulcerations

**FIGURE 4:**
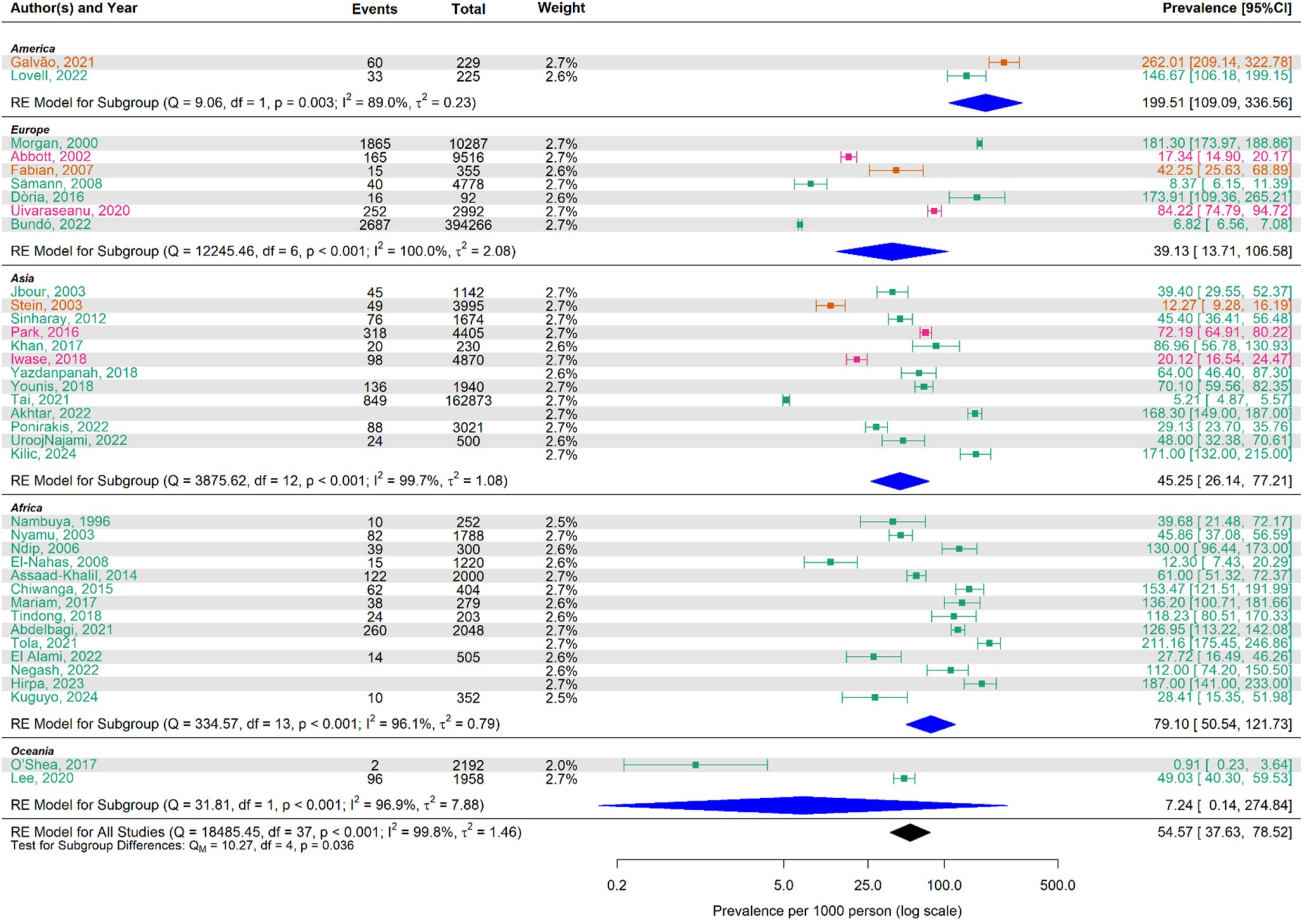
Forest plot showing the estimated prevalence of active ulcerations. Studies are stratified by geographical zone and coloured according to the prevalence type: green for annual prevalence, magenta for period prevalence, and orange for unknown prevalence. The summary polygon below each subgroup (filled in blue) shows the results of a fixed or random‐effects model for just the studies within that group. The summary polygon at the bottom of the plot shows the results of the model when all studies are analysed. The test for subgroup difference (Q_M_) reflects the statistical significance of the subgroup covariate when included in a meta‐regression model. The number of events and the total number of subjects are reported only for studies with available information. I^2^, Inconsistency measure; Q, Cochran's Q statistic, along with degrees of freedom (df) and its *p*‐value; RE, Random‐effects; *τ* ^2^, Between‐study variance.

Thirty‐eight studies contributed to the analysis (Figure 4). The random‐effects pooled prevalence of active ulcerations was 54.57 cases per 1000 persons (95% CI, 37.63–78.52), with substantial heterogeneity (*Q* = 18,485.45; df = 37; *p* < 0.001; I^2^ = 99.8%; *τ* ^2^ = 1.46). When stratified by geographical region, the pooled subgroup prevalence estimates were 199.51 (95% CI, 109.09–336.56) cases per 1000 persons in America, 39.13 (95% CI, 13.71–106.58) in Europe, 45.25 (95% CI, 26.14–77.21) in Asia, 79.10 (95% CI, 50.54–121.73) in Africa, and 7.24 (95% CI, 0.14–274.84) in Oceania.

Visual inspection of the funnel plot revealed no marked asymmetry, with studies clustering in the upper part due to their high precision. However, Begg and Mazumdar's rank correlation test showed a significant negative correlation between effect size and study precision (Kendall's *τ* = −0.23, *p* = 0.039) (Supporting Information S1: Figure S6). This result does not necessarily suggest publication bias but may rather reflect a statistical artefact driven by a highly precise study [40] whose point estimates lie far from the pooled effect. In the meta‐regression analysis (Table 4), length of follow‐up, diabetes duration, study setting, and geographical zone accounted for the largest reductions in between‐study heterogeneity (*R* ^2^ = 31.0%, 29.6%, 16.3% and 12.5%, respectively), despite information about the length of follow‐up being available for only five studies. Among these covariates, only study setting and geographical zone showed a statistically significant non‐zero regression coefficient (Wald test *p* = 0.012 and *p* = 0.036, respectively). Age at enrolment also showed a statistically significant non‐zero regression coefficient (Wald test *p* = 0.030), although it did not account for a meaningful reduction in between‐study heterogeneity. Only the length of follow‐up resulted in a slight shift in the pooled effect estimate (60.09; 95% CI, 25.69–134.17 cases per 1000 persons), noting that this variable was available for only five studies.

**TABLE 4:** Results from meta‐regression for both the prevalence of ulcerations and amputations, along with an assessment of residual heterogeneity.

Outcome	Covariate	Meta‐regression analysis				Heterogeneity assessment	
		*p* ^e^	Cochran's Q (df)	*p* (*Q* test)	Pooled estimate	τ^2^ ^f^	*R* ^2^ (%)^g^
Active ulcerations	None (random‐effects MA)	—	18485.45 (df = 37)	*p* < 0.001	54.57 [37.63, 78.52]	1.46	—
	Study's publication year	*p* = 0.230	10505.53 (df = 36)	*p* < 0.001	54.58 [37.73, 78.32]	1.44	1.5
	Age at enrolment (years)	*p* = 0.030	4134.28 (df = 29)	*p* < 0.001	50.49 [33.74, 74.91]	1.39	5.3
	Sex (% of women)	*p* = 0.950	8091.03 (df = 31)	*p* < 0.001	51.37 [33.59, 77.81]	1.65	0.0
	Diabetes duration (years)	*p* = 0.530	6842.05 (df = 24)	*p* < 0.001	54.81 [37.57, 79.32]	1.03	29.6
	Follow‐up (years)	*p* = 0.120	619.67 (df = 3)	*p* < 0.001	60.09 [25.69, 134.17]	1.01	31.0
	Study period (years)^d^	*p* = 0.130	14622.12 (df = 33)	*p* < 0.001	54.69 [37.66, 78.77]	1.35	7.5
	Study design (retrospective vs. prospective study)	*p* = 0.640	16068.45 (df = 35)	*p* < 0.001	54.58 [37.41, 79.00]	1.51	0.0
	Diabetic foot identification method (diagnostic codes vs. others)	*p* = 0.160	4940.93 (df = 36)	*p* < 0.001	54.70 [37.96, 78.23]	1.41	3.4
	Data source^b^	*p* = 0.120	14344.52 (df = 33)	*p* < 0.001	54.93 [38.03, 78.73]	1.39	4.8
	Setting^c^	*p* = 0.012	11180.56 (df = 34)	*p* < 0.001	54.20 [38.55, 75.72]	1.23	16.3
	Geographical zone^a^	*p* = 0.036	16496.53 (df = 33)	*p* < 0.001	54.20 [38.26, 76.26]	1.28	12.5
	Prevalence type (annual vs. period vs. unknown)	*p* = 0.830	18169.50 (df = 35)	*p* < 0.001	54.56 [37.29, 79.19]	1.54	0.0
Past ulcerations	None (random‐effects MA)	—	1373.83 (df = 19)	*p* < 0.001	72.63 [48.21, 108.02]	0.97	—
	Study's publication year	*p* = 0.670	1373.59 (df = 18)	*p* < 0.001	72.69 [47.80, 109.05]	1.01	0.0
	Age at enrolment (years)	*p* = 0.430	729.64 (df = 14)	*p* < 0.001	76.60 [50.96, 113.59]	0.76	20.9
	Sex (% of women)	*p* = 0.140	896.64 (df = 16)	*p* < 0.001	69.63 [45.14, 105.94]	0.97	0.1
	Diabetes duration (years)	*p* = 0.710	766.76 (df = 13)	*p* < 0.001	80.58 [51.46, 124.02]	0.87	9.7
	Follow‐up (years)	*p* = 0.270	66.23 (df = 1)	*p* < 0.001	62.56 [31.36, 120.93]	0.40	58.3
	Study period (years)^d^	*p* = 0.260	1205.10 (df = 16)	*p* < 0.001	70.58 [44.95, 109.14]	1.05	0.0
	Study design (retrospective vs. prospective study)	*p* = 0.130	1027.34 (df = 17)	*p* < 0.001	72.81 [49.34, 106.19]	0.87	10.0
	Diabetic foot identification method (diagnostic codes vs. others)	N.E.	N.E.	N.E.	N.E.	N.E.	N.E.
	Data source^b^	*p* = 0.560	992.37 (df = 17)	*p* < 0.001	72.52 [47.75, 108.68]	1.00	0.0
	Setting^c^	*p* = 0.800	1318.39 (df = 16)	*p* < 0.001	72.70 [47.12, 110.55]	1.08	0.0
	Geographical zone^a^	*p* = 0.080	580.64 (df = 15)	*p* < 0.001	72.50 [50.21, 103.61]	0.77	19.9
	Prevalence type (annual vs. period vs. unknown)	*p* = 0.150	1028.98 (df = 17)	*p* < 0.001	72.80 [49.19, 106.47]	0.88	8.6
Amputations	None (random‐effects MA)	—	4934.45 (df = 27)	*p* < 0.001	14.75 [9.52, 22.77]	1.35	—
	Study's publication year	*p* = 0.028	2043.80 (df = 26)	*p* < 0.001	14.61 [9.71, 21.93]	1.17	13.4
	Age at enrolment (years)	*p* = 0.640	1981.26 (df = 22)	*p* < 0.001	16.12 [10.00, 25.91]	1.40	0.0
	Sex (% of women)	*p* = 0.930	2781.54 (df = 23)	*p* < 0.001	15.86 [9.95, 25.19]	1.38	0.0
	Diabetes duration (years)	*p* = 0.009	2261.89 (df = 21)	*p* < 0.001	15.09 [9.78, 23.20]	1.07	20.8
	Follow‐up (years)	*p* < 0.001	57.75 (df = 3)	*p* < 0.001	6.46 [3.89, 10.72]	0.32	76.4
	Study period (years)^d^	*p* = 0.670	3443.52 (df = 23)	*p* < 0.001	13.32 [8.21, 21.52]	1.47	0.0
	Study design (retrospective vs. prospective study)	*p* = 0.430	4286.77 (df = 25)	*p* < 0.001	14.83 [9.56, 22.95]	1.37	0.0
	Diabetic foot identification method (diagnostic codes vs. others)	*p* < 0.001	424.48 (df = 26)	*p* < 0.001	15.28 [11.70, 19.93]	0.47	65.6
	Data source^b^	*p* < 0.001	545.52 (df = 24)	*p* < 0.001	14.98 [10.60, 21.13]	0.82	39.2
	Setting^c^	*p* = 0.070	2349.83 (df = 24)	*p* < 0.001	14.87 [9.90, 22.27]	1.16	14.5
	Geographical zone^a^	*p* = 0.290	1849.75 (df = 23)	*p* < 0.001	14.69 [9.58, 22.47]	1.29	4.5
	Prevalence type (annual vs. period vs. unknown)	*p* = 0.140	3398.85 (df = 25)	*p* < 0.001	14.79 [9.69, 22.51]	1.26	6.9

Abbreviations: df, Degrees of freedom referred to the Cochran's Q test; MA, Meta‐analysis; N.E., Not estimable.

^a^ The following geographical regions were considered: America, Europe, Asia, Africa, and Oceania.

^b^ The following types of data sources were considered: primary data collection only, multiple data sources, healthcare data sources, and other databases.

^c^ The following settings were considered: population‐/registry‐based, primary/community care‐based, specialist outpatient clinic‐based, and hospital‐based/inpatient or tertiary care.

^d^ The study period was defined by two study‐level covariates: the calendar year of study start and the study duration, calculated as the difference between the study end year and the study start year. Both covariates were included in the model; however, for cross‐sectional studies reporting annual prevalence only, the study start year alone was included.

^e^ *p*‐value from omnibus Wald‐type test of parameters (i.e., study‐level covariates included in the model).

^f^ Between‐study variance was estimated before (total) and after (residual) inclusion of each study‐level covariate in the model.

^g^ *R* ^2^ is the proportion of the total between‐study variance that is ‘explained’ (i.e., reduced) by the effect of the study‐level covariate included in the model. Negative values are set to zero.

#### 3.4.2 Prevalence of Past Ulcerations

**FIGURE 5:**
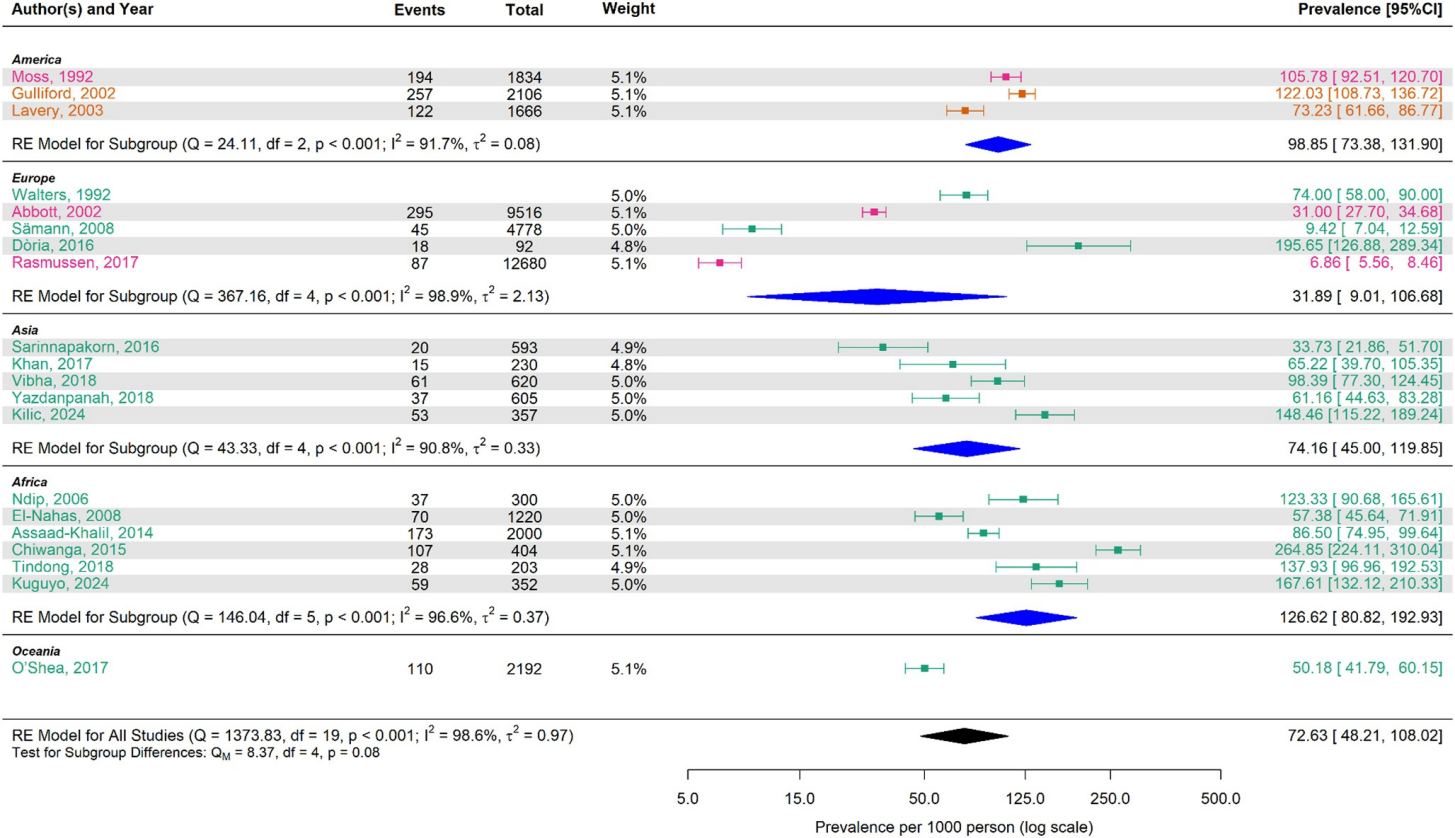
Forest plot showing the estimated prevalence of historical (past) ulcerations. Studies are stratified by geographical zone and coloured according to the prevalence type: green for annual prevalence, magenta for period prevalence, and orange for unknown prevalence. The summary polygon below each subgroup (filled in blue) shows the results of a fixed or random‐effects model for just the studies within that group. The summary polygon at the bottom of the plot shows the results of the model when all studies are analysed. The test for subgroup difference (Q_M_) reflects the statistical significance of the subgroup covariate when included in a meta‐regression model. The number of events and the total number of subjects are reported only for studies with available information. I^2^, Inconsistency measure; Q, Cochran's Q statistic, along with degrees of freedom (df) and its *p*‐value; RE, Random‐effects; *τ* ^2^, Between‐study variance.

Twenty studies contributed to the analysis (Figure 5). The random‐effects pooled prevalence of historical (past) ulcerations was 72.63 (95% CI, 48.21–108.02) cases per 1000 persons, with substantial heterogeneity (*Q* = 1373.83; df = 19; *p* < 0.001; I^2^ = 98.6%; *τ* ^2^ = 0.97). When stratified by geographical region, the pooled subgroup prevalence estimates were 95.85 (95% CI, 73.38–131.90) cases per 1000 persons in America, 31.89 (95% CI, 9.01–106.68) in Europe, 74.16 (95% CI, 45.00–119.85) in Asia, and 126.62 (95% CI, 80.82–192.93) in Africa. As for Oceania, only one study was included in the meta‐analysis, reporting a prevalence of 50.18 (95% CI, 41.79–60.15) cases per 1000 persons.

Visual inspection of the funnel plot revealed no marked asymmetry, and Begg and Mazumdar's rank correlation test showed no significant correlation between effect size and study precision (Kendall's *τ* = −0.02, *p* = 0.92) (Supporting Information S1: Figure S7). In the meta‐regression analysis (Table 4), length of follow‐up, age at enrolment, and geographical zone accounted for the largest reductions in between‐study heterogeneity (*R* ^2^ = 58.3%, 20.9%, and 19.9%, respectively). None of these covariates showed a statistically significant non‐zero regression coefficient. The study setting showed a statistically significant non‐zero regression coefficient (Wald tests *p* < 0.001), although it did not account for a meaningful reduction in between‐study heterogeneity. Only length of follow‐up resulted in a substantial shift in the pooled effect estimate (62.56; 95% CI, 31.36–120.93 cases per 1000 persons), noting that this variable was available for only three studies.

#### 3.4.3 Prevalence of Amputations

**FIGURE 6:**
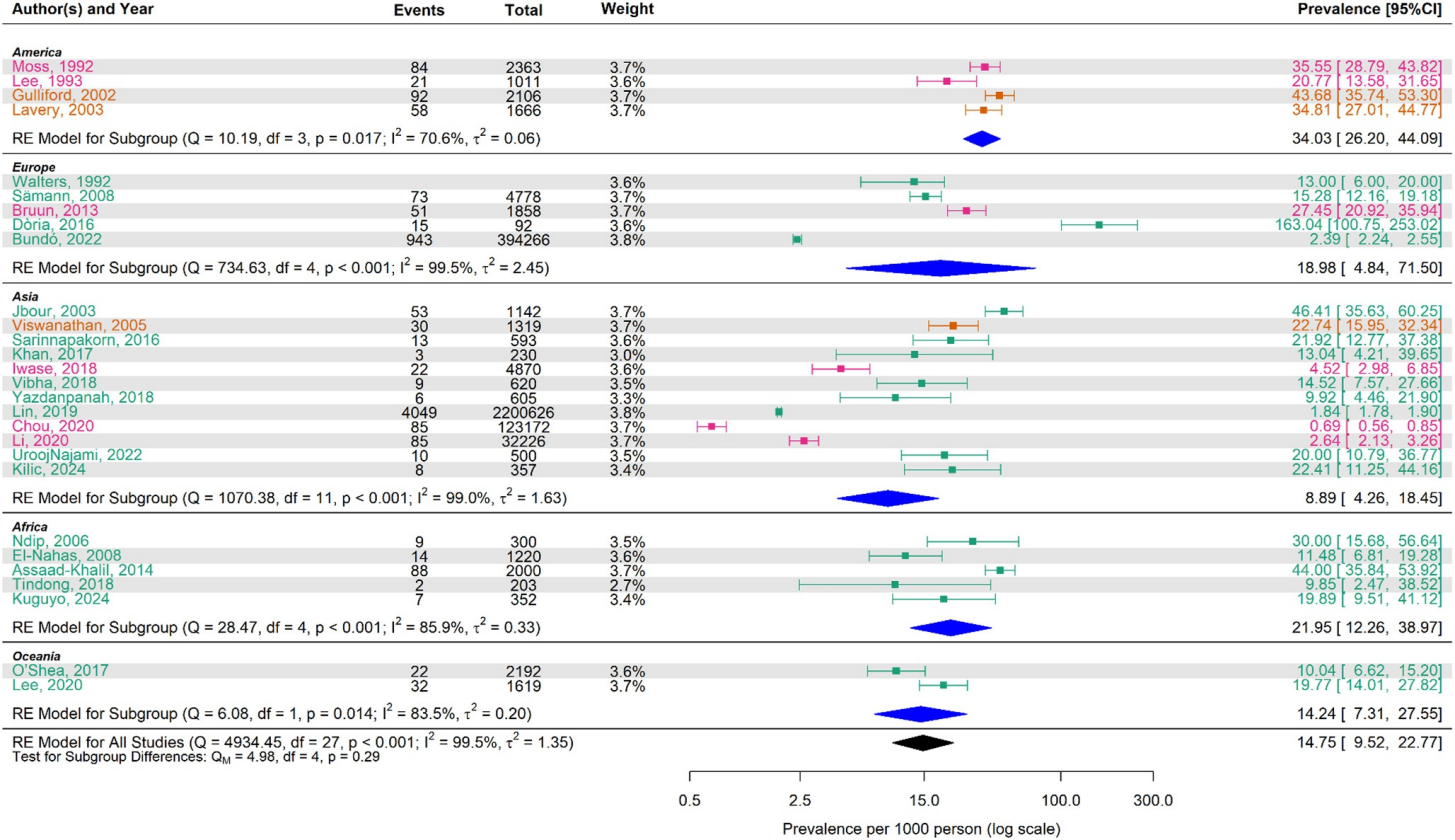
Forest plot showing the estimated prevalence of amputations. Studies are stratified by geographical zone and coloured according to the prevalence type: green for annual prevalence, magenta for period prevalence, and orange for unknown prevalence. The summary polygon below each subgroup (filled in blue) shows the results of a fixed or random‐effects model for just the studies within that group. The summary polygon at the bottom of the plot shows the results of the model when all studies are analysed. The test for subgroup difference (Q_M_) reflects the statistical significance of the subgroup covariate when included in a meta‐regression model. The number of events and the total number of subjects are reported only for studies with available information. I^2^, Inconsistency measure; Q, Cochran's Q statistic, along with degrees of freedom (df) and its *p*‐value; RE, Random‐effects; *τ* ^2^, Between‐study variance.

Twenty‐eight studies contributed to the analysis (Figure 6). The random‐effects pooled prevalence of amputations was 14.75 (95% CI, 9.52–22.77) cases per 1000 persons, with substantial heterogeneity (*Q* = 4934.45; df = 27; *p* < 0.001; I^2^ = 99.5%; *τ* ^2^ = 1.35). When stratified by geographical region, the pooled subgroup prevalence estimates were 34.03 (95% CI, 26.20–44.09) cases per 1000 persons in America, 18.98 (95% CI, 4.84–71.50) in Europe, 8.89 (95% CI, 4.26–18.45) in Asia, 21.95 (95% CI, 12.26–38.97) in Africa, and 14.24 (95% CI, 7.31–27.55) in Oceania.

Visual inspection of the funnel plot revealed no marked asymmetry, and Begg and Mazumdar's rank correlation test showed no significant correlation between effect size and study precision (Kendall's *τ* = −0.25, *p* = 0.06) (Supporting Information S1: Figure S8). In the meta‐regression analysis (Table 4), length of follow‐up, DF identification method, study data source, diabetes duration, study setting, and publication year accounted for the largest reductions in between‐study heterogeneity (*R* ^2^ = 76.4%, 65.6%, 39.2%, 20.8%, 14.5%, and 13.4%, respectively). All these covariates were associated with the study mean estimates (i.e., at study‐level data), showing statistically significant non‐zero regression coefficients, with the exception of the study setting. Among them, only length of follow‐up also produced a meaningful shift in the pooled effect estimate (6.46 cases per 1000 persons; 95% CI, 3.89–10.72), although this variable was available in only five studies. Forest plots stratified using the DF identification method and type of data source are shown in Supporting Information S1: Figure S9 and S10, respectively.

#### 3.4.4 Prevalence of Infections

The forest plot in Supporting Information S1: Figure S11 summarises the prevalence of DF‐related infections. Only 4 studies were included, yielding a random‐effects pooled prevalence of DF infections of 39.17 (95% CI, 6.81–195.07) cases per 1000 persons. Again, heterogeneity was substantial (*Q* = 1767.13, df = 3, *p* < 0.001; I^2^ = 99.8%; *τ* ^2^ = 3.30), largely reflecting the marked divergence in prevalence estimates across studies, which limits the interpretability of the pooled estimate.

### 3.5 Risk of Bias Assessment

Of the 18 prospective cohort studies included in this systematic review, 2 (11.1%) were considered at ‘moderate’ risk of bias, and the others (*N* = 16, 88.9%) were rated as at ‘low’ risk of bias (Supporting Information S1: Table S3). Among the 33 retrospective cohort studies included, the risk of bias was considered ‘moderate’ for 20 (60.6%), and ‘low’ for the remaining 13 studies (39.4%) (Supporting Information S1: Table S4). No cohort study was rated as having a ‘high’ risk of bias, and the majority of biases identified were related to the identification of confounders and the strategies used to deal with these confounders, including whether appropriate statistical analyses were used to evaluate the risk factors associated with the development of DF disease.

Among the 38 cross‐sectional studies, 3 studies (7.9%) were considered at ‘moderate’ risk, and the remaining 35 studies (92.1%) at ‘low’ risk of bias (Supporting Information S1: Table S5). Most biases were related to the description of the setting and population under study.

## 4 Discussion

This systematic review and meta‐analysis provides a comprehensive overview of the global epidemiology of DF disease, encompassing ulcerations, amputations, and infections. Many of the primary studies included in our systematic review found that known risk factors for DF disease, such as prior ulceration and amputation, peripheral neuropathy (e.g., insensitivity to monofilament), peripheral arterial disease, foot deformities, age and longer diabetes duration, are key predictors of new ulceration or amputation [18, 31, 32, 33, 35, 36, 37, 38, 40, 43, 44, 46, 47, 53, 54, 57, 60, 66, 67, 70, 72, 73, 80, 81, 82, 84, 85, 89, 91, 98, 99, 100, 103, 106, 108, 112, 113, 116]. Epidemiological estimates of our study confirm that DF disease is common and clinically relevant in individuals with DM, with pooled incidence and prevalence estimates that underscore the magnitude of the problem worldwide. However, these estimates should be interpreted with caution, given the substantial heterogeneity observed across studies (I^2^ > 90%), as they did not represent a single common underlying effect. Instead, these estimates offer a statistical summary across various study contexts rather than globally representative values. The primary sources of heterogeneity were the length of follow‐up, geographical zone, publication year, and diabetes duration. Moreover, the study period significantly reduced the between‐study variance concerning the incidence of ulceration, while age at enrolment significantly reduced the between‐study variance concerning the prevalence of past ulceration. Both DF identification and the study data source significantly reduced the between‐study variance concerning the prevalence of amputation.

Overall, the meta‐regression analyses suggested that several study‐level characteristics were associated with reductions in between‐study heterogeneity, both for incidence and prevalence outcomes. These associations may reflect differences in study design, population characteristics, outcome ascertainment, and data structure across studies. The inclusion of community‐based, general‐population, outpatient, hospital‐based, and inpatient populations also contributed to an increase in the statistical heterogeneity observed in the pooled estimates.

As for diabetes duration, it was identified as a significant risk factor for ulcers, infections, and/or amputation in numerous studies [18, 32, 34, 36, 40, 54, 59, 60, 65, 67, 73, 75, 80, 84, 86, 89, 92, 100, 103, 104, 106, 108, 110, 113, 114, 115]. Notably, DF disease can also be present at the initial diagnosis of DM, as highlighted by specific studies conducted at DM onset, that is, Nambuya et al. (1996) [79], Sinharay et al. (2012) [19], Chou et al. (2020) [42], and Rossboth et al. (2021) [116]. These studies generally reported lower, but non‐zero, epidemiological rates compared to research on patients with long‐standing diabetes, supporting the need for starting DF screening at DM diagnosis.

Regarding geographical zone, epidemiological estimates of DF disease in America and Africa were above the pooled global estimates, whereas those in Europe, Asia, and Oceania generally fell below the average. Notably, the relatively lower estimates observed in Europe are striking when considered in light of the older age structure of its population and the consequently longer duration of diabetes. This pattern raises the hypothesis that differences in the organization and quality of diabetes care may contribute to mitigating the incidence of DF disease in Europe compared with other regions [118, 119]. However, drawing broad generalizations from a continental perspective is inherently challenging because of the potential influence of detection and temporal biases (i.e., diagnostic and therapeutic approaches vary substantially across countries and have evolved over time) and, more importantly, because each continent encompasses populations with markedly heterogeneous sociocultural and healthcare contexts. As a pragmatic example of intra‐regional differences, in our meta‐analysis of amputation incidence rates, American data included the study by Nelson et al. (1988) [84], based on standardized physical and laboratory examinations in a population of Pima Indians, as well as the study by Déruaz‐Luyet et al. (2020) [45], which analysed insurance claims from the IBM MarketScan Research Database in the general United States population. The two studies differ considerably in terms of study period, study population, and data sources, and found widely divergent incidence rates (10.36 cases per 1000 person‐years [95% CI, 8.32–12.90] and 1.90 cases per 1000 person‐years [95% CI, 1.87–1.93], respectively).

Almost one‐third of the included studies evaluated the epidemiology of DF disease using real‐world data sources (e.g., claims databases, electronic health records, and diabetes registries). The primary advantage of real‐world data over other sources is their ability to capture large populations. Nevertheless, important limitations of real‐world data must be acknowledged. One of the most critical challenges is the accurate identification of the condition of interest, which relies on the availability of specific diagnostic codes (e.g., ICD‐9 or ICD‐10). Furthermore, because claims data are mainly collected for administrative purposes, the estimation of epidemiological parameters may vary across populations depending on administrative and coding practices.

The high burden of DF conditions documented by this systematic review confirms that DF disease remains a major public health concern worldwide [4]. These conditions are not only common but also extremely debilitating and potentially fatal. The prognosis for patients who undergo a foot amputation is generally poor, with overall 5‐year mortality rates varying from 29% to 69% following minor amputations, and from 52% to 80% for patients with major amputations [120, 121]. Additionally, the related loss of mobility and independence associated with foot ulcers and amputations significantly impacts the quality of life [1, 7]. Lastly, DF disease exerts a substantial economic burden on healthcare systems [1, 2, 6, 7, 122]. Indeed, it is estimated that up to one‐third of all DM‐related healthcare expenditures may be attributable to DF conditions, including costs of prolonged wound care, surgeries, prosthetics, rehabilitation, and treatment of infections [122, 123]. Indirect costs should also be acknowledged, as DF disease is associated with loss of productivity, underemployment/unemployment, absenteeism, reduced family assets, early retirement, transportation, psychotherapy, and home care [122].

### 4.1 Limitations

This review has several limitations that need to be acknowledged. First, the evidence base was heterogeneous and subject to potential biases. We included studies from a wide range of settings with varying designs (cohort and cross‐sectional) and definitions of DF disease, which contributed to the high between‐study heterogeneity in these meta‐analyses. However, analyses were stratified by DF condition as defined by the study authors (i.e., ulceration, amputation, and infection) and we explored the primary pre‐identified sources of variability via meta‐regression. Second, there is a lack of standardisation in how DF outcomes are defined and reported across studies [4, 124]. For example, the definition of ‘diabetic foot infection’ and ‘lower extremity amputation’ considerably differed in severity or scope across the included studies, as demonstrated also by different codes searched in claims databases and clinical registries. Third, despite the use of multiple databases and citation searching of relevant systematic reviews and meta‐analyses, some eligible studies may not have been captured. This may have occurred particularly for studies indexed using narrower or alternative terminology for specific outcomes, such as DF infection, ischaemia, or diabetes‐related amputation, or for studies in which DF outcomes were not the primary focus. Fourth, data gaps remain for certain regions and populations. Relatively few studies were found in Latin America and Oceania and in low‐income countries, reflecting the well‐known paucity of data on DF outcomes in these areas [12]. Restricting eligible studies to those published in English may have further excluded relevant data from non‐English‐speaking countries, potentially introducing language bias [125]. Fifth, separate analyses of epidemiologic measures for type 1 and type 2 diabetes could not be conducted because of insufficient disaggregated data. Sixth, the pooled estimate for infection incidence was based on only two studies. Although a narrative synthesis may have been more appropriate for this outcome, a meta‐analytic estimate was retained to ensure a consistent analytical framework across all evaluated outcomes. Therefore, this estimate should be considered exploratory and interpreted with particular caution. Lastly, many of the included studies (especially retrospective studies) did not report or analyse how potential confounders or comorbidities were accounted for; as noted in the risk‐of‐bias assessment, some cohort studies did not (or did not adequately) adjust for differences in patient risk factors when reporting incidence or prevalence. This could lead to over‐ or underestimation of the true rates.

## 5 Conclusion

In conclusion, this study provides robust aggregated estimates and confirms that DF disease is a common and serious consequence of DM worldwide. Our findings reinforce the urgent need for continued investment in preventive foot care, patient education, and health system strategies (such as multidisciplinary foot care teams and improved access to podiatric and vascular services) to reduce the occurrence of DF ulcers, amputations, and infections. Importantly, the epidemiological indicators and insights into data source provided here may support future clinical and pharmacoepidemiological studies, for example, studies evaluating new interventions or monitoring temporal trends using large‐scale data.

## Author Contributions

G.T. supervised the study and conceived the concept. M.C., S.C., F.M., D.P., and A.Fr. conducted the first phases of the review (i.e., preliminary searches, study selection process, formal screening of search results against eligibility criteria, data extraction, and risk of bias assessment). A.Fo. performed the statistical analyses and was responsible for the statistical methods and results sections. M.C. wrote the first draft of the manuscript. Y.I., S.C., E.M., and G.T. critically revised the manuscript. All authors made important intellectual contributions and approved the manuscript for submission. The corresponding author (G.T.) attests that all listed authors meet authorship criteria and that no others meeting the criteria have been omitted.

## Funding

The study received an unconditional grant from Fondazione Alitti O.N.L.U.S. The sponsor did not influence the investigators in any phase of the study.

## Conflicts of Interest

G.T. has served, over the last 3 years, on advisory boards/seminars funded by Sanofi, MSD, Eli Lilly, Sobi, Celgene, Daiichi Sankyo, Novo Nordisk, Gilead, and Amgen on topics not related to the content of this paper; he is also a scientific coordinator of the academic spin‐off ‘INSPIRE srl,’ which has received funding for conducting observational studies, systematic reviews, and research activities from contract research organizations (RTI Health Solutions, Pharmo Institute N.V.) and from Pharmaceutical Companies (Chiesi Italia, Kyowa Kirin srl, Daiichi Sankyo Italia S.p.A., Grunenthal, Novo Nordisk, Shionogi, and Sanofi). Additionally, he is currently a consultant for Viatris in a legal case concerning an adverse reaction to sertraline. None of these listed activities is related to the topic of the article. Y.I. participated in seminars as a lecturer on topics not related to the paper and sponsored by the following pharmaceutical companies in the last 2 years: AstraZeneca, Sandoz SpA. She is the CEO of the academic spin‐off ‘INSPIRE srl.’ The other authors have no relevant affiliations or financial involvement with any organization or entity with a financial interest in or financial conflict with the subject matter or materials discussed in the manuscript. This includes employment, consultancies, honoraria, stock ownership or options, expert testimony, grants or patents received or pending, or royalties.

## Supporting information


Supporting Information S1

## Data Availability

The data that support the findings of this study are available from the corresponding author upon reasonable request.
