# Supplementary material for: Global Epidemiology of Diabetic Foot: A Systematic Review and Meta‐Analysis
Source: Diabetes Metab Res Rev. 2026 Aug 31;42(6):e70223. doi: 10.1002/dmrr.70223 (PMC13528280; doi:10.1002/dmrr.70223)
Supplement: Supplementary file 1 [file DMRR-42-e70223-s001.docx]

**SUPPLEMENTARY MATERIAL**

**Title:** Global Epidemiology of Diabetic Foot: A Systematic Review and Meta-analysis

**Authors:**

Massimo Carollo, MD^1^; Ylenia Ingrasciotta, PhD^1,2^; Salvatore Crisafulli, PhD^1,2^; Francesco Maccarrone, MSc^1^; Daniela Pellegrino, MSc^1^; Alberto Francescon, MSc^1^; Edoardo Mannucci, MD^3,4^; Andrea Fontana, MSc^5^; Gianluca Trifirò, PhD^1,2^**^*^**

**Affiliations:**

1. Department of Diagnostics and Public Health, University of Verona, Verona, Italy
2. Academic Spin-off “Innovative Solutions for Medical Prediction and Big Data Integration in Real World Setting Srl—INSPIRE SRL”, University of Messina, Messina, Italy
3. Department of Biomedical, Experimental and Clinical Sciences, University of Florence, Florence, Italy
4. Fondazione Alitti O.N.L.U.S., Florence, Italy
5. Unit of Biostatistics, Fondazione IRCCS Casa Sollievo della Sofferenza, San Giovanni Rotondo, Foggia, Italy

**ORCIDs:**

**Massimo Carollo**: 0000-0002-6523-6036

**Ylenia Ingrasciotta**: 0000-0002-9549-6111

**Salvatore Crisafulli**: 0000-0001-6625-1943

**Francesco Maccarrone**: 0009-0005-5344-5278

**Daniela Pellegrino**: 0009-0008-1573-4345

**Alberto Francescon**: 0009-0003-3192-859X

**Andrea Fontana**: 0000-0002-6660-5315

**Edoardo Mannucci**: 0000-0001-9759-9408

**Gianluca Trifirò**: 0000-0003-1147-7296

***Corresponding author**

Prof. Gianluca Trifirò

gianluca.trifiro@univr.it

Department of Diagnostics and Public Health, University of Verona, Verona, Italy

P.le L.A. Scuro 10, 37124, Verona

**Table of contents**

[**Table 1**. Search strategy.	3](#_Toc234662814)

[**Table 2**. Excluded studies after full-text assessment for eligibility.	5](#_Toc234662815)

[**Table 3**. Risk of bias of prospective cohort studies included in the systematic review.	11](#_Toc234662816)

[**Table 4**. Risk of bias of retrospective cohort studies included in the systematic review.	12](#_Toc234662817)

[**Table 5**. Risk of bias of cross-sectional studies included in the systematic review.	14](#_Toc234662818)

[**Figure 1**. Funnel plot of the incidence of ulcerations with estimated Kendall's tau coefficient to test for asymmetry that may cause publication bias.	16](#_Toc234662819)

[**Figure 2**. Funnel plot of the incidence of ulcerations, including imputed studies following the application of the Trim and Fill method.	17](#_Toc234662820)

[**Figure 3**. Funnel plot of the incidence of ulcerations, having excluded the following two highly precise studies: Lu CW et al. (2021) and Lovell L et al. (2023).	18](#_Toc234662821)

[**Figure 4**. Funnel plot of the incidence of amputations with estimated Kendall's tau coefficient to test for asymmetry that may cause publication bias.	19](#_Toc234662822)

[**Figure 5**. Forest plot showing the estimated incidence of infections.	20](#_Toc234662823)

[**Figure 6**. Funnel plot of the prevalence of active ulcerations with estimated Kendall's tau coefficient to test for asymmetry that may cause publication bias.	21](#_Toc234662824)

[**Figure 7**. Funnel plot of the prevalence of historical (past) ulcerations with estimated Kendall's tau coefficient to test for asymmetry that may cause publication bias.	22](#_Toc234662825)

[**Figure 8**. Funnel plot of the prevalence of amputations with estimated Kendall's tau coefficient to test for asymmetry that may cause publication bias.	23](#_Toc234662826)

[**Figure 9**. Forest plot showing the estimated prevalence of amputations, stratified by diabetic foot identification method (i.e., diagnostic codes vs. other codes).	24](#_Toc234662827)

[**Figure 10**. Forest plot showing the estimated prevalence of amputations, stratified by study data source type.	25](#_Toc234662828)

[**Figure 11**. Forest plot showing the estimated prevalence of infections.	26](#_Toc234662829)

# **Table 1**. Search strategy.

| **Query** | **PubMed** | **Results** |
| --- | --- | --- |
| #1 | "Diabetic Foot"[MeSH Terms] OR "diabetic foot"[Title/Abstract] OR "diabetic feet"[Title/Abstract] | 17,519 |
| #2 | "Foot Ulcer"[MeSH Terms] OR "Foot Diseases"[MeSH Terms] OR “amputation surg*” [Title] OR "lower extremity complication*"[Title] OR "foot condition*"[Title] OR "feet condition*"[Title] OR "foot complication*"[Title] OR “feet complication*” [Title] OR "foot ulcer*"[Title] OR "feet ulcer*"[Title] OR "foot disease*"[Title] OR "feet disease*"[Title] OR "macrovascular complication*"[Title] | 36,353 |
| #3 | "Diabetes Mellitus"[MeSH Terms] OR "diabetes"[Title] | 589,363 |
| #4 | #2 AND #3 | 14,458 |
| #5 | #1 OR #4 | 18,883 |
| #6 | "Epidemiology"[MeSH Terms] OR "epidemiolog*"[Title] OR "Prevalence"[MeSH Terms] OR "prevalence"[Title] OR "Incidence"[MeSH Terms] OR "incidence"[Title] OR "cohort"[Title] | 1,052,105 |
| #7 | #5 AND #6 | 1,339 |
| **Query** | **EMBASE** | **Results** |
| #1 | diabetic foot'/exp OR 'diabetic foot':ti,ab OR 'diabetic feet':ti,ab | 26,164 |
| #2 | foot ulcer'/mj OR 'foot disease'/mj OR 'amputation surg*':ti OR 'lower extremity complication*':ti OR 'foot condition*':ti OR 'feet condition*':ti OR 'foot complication*':ti OR 'feet complication*':ti OR 'foot ulcer*':ti OR 'feet ulcer*':ti OR 'foot disease*':ti OR 'feet disease*':ti OR 'macrovascular complication*':ti | 17,213 |
| #3 | diabetes mellitus'/mj OR 'diabetes':ti | 546,330 |
| #4 | #2 AND #3 | 2,617 |
| #5 | #1 OR #4 | 27,077 |
| #6 | epidemiology'/mj OR 'epidemiolog*':ti OR 'prevalence'/mj OR 'prevalence':ti OR 'incidence'/mj OR 'incidence':ti OR 'cohort':ti | 883,239 |
| #7 | #5 AND #6 | 1,085 |
| **Query** | **CINAHL Plus** | **Results** |
| #1 | SU "diabetic foot" OR TI ( "diabetic foot" OR "diabetic feet" ) OR AB ( "diabetic foot" OR "diabetic feet" ) | 12,453 |
| #2 | SU ( "foot ulcer" OR "foot disease" ) OR ( "amputation surg*" OR "lower extremity complication*" OR "foot condition*" OR "feet condition*" OR "foot complication*" OR "feet complication*" OR "foot ulcer*" OR "feet ulcer*" OR "foot disease*" OR "feet disease*" OR "macrovascular complication*" ) | 11,348 |
| #3 | SU diabetes mellitus OR TI diabetes | 207,250 |
| #4 | #2 AND #3 | 3,368 |
| #5 | #1 OR #4 | 3,368 |
| #6 | SU ( epidemiology OR prevalence OR incidence ) OR TI ( epidemiolog* OR prevalence OR incidence OR cohort ) | 650,851 |
| #7 | #5 AND #6 | 665 |
| **Query** | **Cochrane Library** | **Results** |
| #1 | MeSH descriptor: [Diabetic Foot] explode all trees | 1,601 |
| #2 | (diabetic foot):ti | 2,749 |
| #3 | (diabetic feet):ti | 2,749 |
| #4 | #1 OR #2 OR #3 | 3,267 |
| #5 | MeSH descriptor: [Foot Ulcer] explode all trees | 1,704 |
| #6 | MeSH descriptor: [Foot Diseases] explode all trees | 1,882 |
| #7 | (amputation surg*):ti | 33 |
| #8 | (lower extremity complication*):ti | 17 |
| #9 | (foot condition*):ti | 9 |
| #10 | (feet condition*):ti | 9 |
| #11 | (foot complication*):ti | 24 |
| #12 | (feet complication*):ti | 24 |
| #13 | (foot ulcer*):ti | 1,915 |
| #14 | (feet ulcer*):ti | 1,915 |
| #15 | (foot disease*):ti | 223 |
| #16 | (feet disease*):ti | 223 |
| #17 | (macrovascular complication*):ti | 33 |
| #18 | #5 OR #6 OR #7 OR #8 OR #9 OR #10 OR #11 OR #12 OR #13 OR #14 OR #15 OR #16 OR #17 | 4,221 |
| #19 | MeSH descriptor: [Diabetes Complications] explode all trees | 10,100 |
| #20 | (diabetes):ti | 51,870 |
| #21 | #19 OR #20 | 59,107 |
| #22 | #18 AND #21 | 1,770 |
| #23 | #4 OR #22 | 3,410 |
| #24 | MeSH descriptor: [Epidemiology] explode all trees | 104 |
| #25 | (epidemiolog*):ti | 1,302 |
| #26 | MeSH descriptor: [Prevalence] explode all trees | 7,470 |
| #27 | (prevalence):ti | 3,523 |
| #28 | MeSH descriptor: [Incidence] explode all trees | 14,558 |
| #29 | (incidence):ti | 8,905 |
| #30 | (cohort):ti | 7,087 |
| #31 | #24 OR #25 OR #26 OR #27 OR #28 OR #29 OR #30 | 37,133 |
| #32 | #23 AND #31 | 51 |

# **Table 2**. Excluded studies after full-text assessment for eligibility.

| **Study** | **Reference** | **Reason(s) for exclusion** |
| --- | --- | --- |
| Aalaa, 2024 | Aalaa M, Vahdani AM, Mohajeri Tehrani M, et al. Epidemiological Insights into Diabetic Foot Amputation and its Correlates: A Provincial Study. Clin Med Insights Endocrinol Diabetes. 2024;17:11795514241227618. Published 2024 Jan 30. doi:10.1177/11795514241227618 | No data on the prevalence or incidence of diabetic foot disease were available. It was only possible to determine the prevalence of diabetic foot amputations in a cohort of patients with foot ulcer |
| Abbas, 2005 | Abbas ZG, Archibald LK. Epidemiology of the diabetic foot in Africa. Med Sci Monit. 2005;11(8):RA262-RA270. | Literature review |
| Brekelmans, 2019 | Brekelmans W, Borger van der Burg BLS, Vroom MA, Kreuger MJ, Schrander van der Meer AM, Hoencamp R. Prevalence of foot ulcers in dialysis-dependent patients. Wound Repair Regen. 2019;27(6):687-692. doi:10.1111/wrr.12750 | Prevalence of foot ulcers in dialysis-dependent patients (both diabetics and non-diabetics) |
| Carro, 2023 | Carro GV, Saurral RN, Witman EL, et al. Clinical features and outcomes of diabetic foot in Argentina: a longitudinal multicenter study. Características clínicas y evolución de pacientes con pie diabético en Argentina: estudio longitudinal multicéntrico. Medicina (B Aires). 2023;83(3):428-441. | No data on the prevalence or incidence of diabetic foot disease were available. Only patients with a new diagnosis of diabetic foot were included in the analysis, with no information on the population at risk |
| Dedov, 2017 | Dedov II, Shestakova MV, Vikulova OK. Epidemiology of diabetes mellitus in the Russian Federation: clinical and statistical report for 2016 according to the federal diabetes registry. Diabetes Mellitus. 2017;20(1):13-41. doi:10.14341/DM8664. | The data reported have already been included in another study (Galstyn, 2018), which is part of this review |
| Duarte, 2009 | Duarte R, Costa AL, Valongo A, Lessa I, Recto A, Oliveira R, Castela A, Boavida JM, Raposo J. Prevalence of risk factors in the diabetic foot ulcer. Rev Port Diabetes. 2009;4(4):180-190. | Conference abstract |
| Ehsanpour, 2015 | Ehsanpour A, Momeni M. An epidemiological study on diabetic foot ulcer patients hospitalized in Razi Hospital (Ahvaz) during 2007-2011. Biomed Pharmacol J. 2015;8(2):1157-1164. doi:10.13005/bpj/871 | No data on the prevalence or incidence of diabetic foot disease were available. It was only possible to determine the percentage of amputated patients within the cohort of patients with diabetic foot ulcer |
| Engberg, 2019 | Engberg S, Kirketerp-Møller K, Ullits Andersen H, Rasmussen A. Incidence and predictors of recurrent and other new diabetic foot ulcers: a retrospective cohort study. Diabet Med. 2019;36(11):1417-1423. doi:10.1111/dme.13964 | No data on the prevalence or incidence of diabetic foot disease were available. Study cohort was patients with a healed diabetic foot ulcer and study objective was to estimate progression rates and associated risk factors |
| Font-Jiménez, 2016 | Font-Jiménez I, Llaurado-Serra M, Roig-Garcia M, De Los Mozos-Perez B, Acebedo-Urdiales S. Retrospective study of the evolution of the incidence of non-traumatic lower-extremity amputations (2007-2013) and risk factors of reamputation. Prim Care Diabetes. 2016;10(6):434-441. doi:10.1016/j.pcd.2016.04.001 | No data on the prevalence or incidence of diabetic foot disease were available. It was only possible to determine the percentage of patients with diabetes within the cohort of amputated patients |
| Guarnotta, 2021 | Guarnotta V, Radellini S, Vigneri E, et al. Diabetic foot ulcers: Retrospective comparative analysis from Sicily between two eras. PLoS One. 2021;16(12):e0259405. Published 2021 Dec 7. doi:10.1371/journal.pone.0259405 | No data on the prevalence or incidence of diabetic foot disease were available |
| Halimi, 1993 | Halimi S, Benhamou PY, Charras H. Incidence and cost of the diabetic foot in France: first data [Le coût du pied diabétique]. Diabete Metab. 1993;19(5 BIS):518-527 | Conference abstract |
| Holman, 2014 | Holman N, Chadwick P, McAdam F, Haycocks S, Young B. A longitudinal cohort of people presenting with diabetic foot ulcers in northern England [ePoster #1147]. Presented at: EASD Annual Meeting; September 17, 2014; Vienna, Austria. Session: PS 100 Management of foot ulcers. Available at: https://easd.org/media-centre/#. Accessed February 5, 2025. | Conference abstract |
| Jorgensen, 2013 | Jorgensen ME, Almdal TP, Færch K. Reduced incidence of lower-extremity amputations in a Danish diabetes population from 2000 to 2012 [ePoster #1251]. Presented at: EASD Annual Meeting; 2013; Barcelona, Spain. Steno Diabetes Center, Denmark. Session: PS 108 Diabetic foot: epidemiology, risk factors, and outcomes. Available at: https://easd.org/media-centre/#. Accessed February 5, 2025 | Conference abstract |
| Kerr, 2019 | Kerr M, Barron E, Chadwick P, et al. The cost of diabetic foot ulcers and amputations to the National Health Service in England. Diabet Med. 2019;36(8):995-1002. doi:10.1111/dme.13973 | No data on the prevalence or incidence of diabetic foot disease were available |
| Koutsovasilis, 2014 | Koutsovasilis A, Papazafiropoulou A, Sotiropoulos A, Bachlitzanaki M, Tamvakos E, Bousboulas S. Differences between rural and urban population considering prevalence of chronic complications among patients with type 2 diabetes mellitus. Diabetes Res Clin Pract. 2014;235(2):E295-E296. | Conference abstract |
| Lawee, 1992 | Lawee D, Csima A. Diabetes-related lower extremity amputations in Ontario: 1987-88 experience. Can J Public Health. 1992;83(4):298-302. | No data were available on the prevalence or incidence of diabetic foot disease, and the diabetic population was estimated based on data from a different geographical region |
| Lawrence, 2004 | Lawrence SM, Wraight PR, Campbell DA, Colman PG. Assessment and management of inpatients with acute diabetes-related foot complications: room for improvement. Intern Med J. 2004;34(5):229-233. doi:10.1111/j.1444-0903.2004.00590.x | No data on the prevalence or incidence of diabetic foot disease were available |
| Lauterbach, 2009 | Lauterbach S, Kostev K, Kohlmann T, Schröder-Bernhardi D. PDB11 database analysis on prevalence and risk factors of diabetic foot syndrome (DFS) in Germany. Value Health. 2009;12(7):A295. doi:10.1016/S1098-3015(10)74989-5. | Conference abstract |
| Lauterbach, 2010 | Lauterbach S. High prevalence of limb amputation in people with diabetic foot syndrome. Diabetologia. 2010;53(Suppl 1):S1-S556. doi:10.1007/s00125-010-1872-z. | Conference abstract |
| Lavery, 2018 | Lavery L, Bloom J, Petersen B, Rothenberg G. Uncensored incidence of diabetic foot ulcers to patients in remission [Abstract #963]. Presented at: EASD Annual Meeting; October 3, 2018; Berlin, Germany. Session: Foot ulcers: morbidity and mortality. Available at: https://easd.org/media-centre/#. Accessed February 5, 2025. | Conference abstract |
| Li, 2011 | Li X, Xiao T, Wang Y, et al. Incidence, risk factors for amputation among patients with diabetic foot ulcer in a Chinese tertiary hospital. Diabetes Res Clin Pract. 2011;93(1):26-30. doi:10.1016/j.diabres.2011.03.014 | No data on the prevalence or incidence of diabetic foot disease were available. It was only possible to determine the prevalence of diabetic foot amputations in a cohort of patients with foot ulcer |
| Mahapatra, 2017 | Mahapatra H, Khuntia M, Sahoo AK, Kanwar JB, Mishra S, Jena AR. Prevalence and risk factors of peripheral arterial disease in subjects with diabetic foot ulcer in Eastern India. Indian J Endocrinol Metab. 2017;21(Suppl 2):S4-S79. | Conference abstract |
| Mohammadi, 2013 | Mohammadi A, Boddington L, King L, Rogers M, Levy JC, Hands L, Byren I, Berendt T, Lipsky B, Tan GD. A prospective audit of diabetic foot care among hospital inpatients: disease prevalence and quality of care. Diabet Med. 2013;30(Suppl 1):30-201. | Conference abstract |
| Nagathan, 2024 | Nagathan V, Yadahalli P, Singaraddi R. Study of incidence of amputation in patients with diabetic foot ulcer. Res J Med Sci. 2024;6(2):124-126. doi:10.36478/10.59218/makrjms.2024.6.124.126 | No data on the prevalence or incidence of diabetic foot disease were available. It was only possible to determine the percentage of amputated patients within the cohort of patients with diabetic foot ulcer |
| Nogueira Smanioto, 2014 | Nogueira Smanioto F, Haddad MCL, Rossaneis MA. Self-care into the risk factors in diabetic foot ulceration: a cross-sectional study. Online Braz J Nurs. 2014;13(3):343-352 | No data on the prevalence or incidence of diabetic foot disease were available. The study focus was on ulceration risk |
| Ogurtsova, 2020 | Ogurtsova K, Morbach S, Haastert B, et al. Cumulative long-term recurrence of diabetic foot ulcers in two cohorts from centres in Germany and the Czech Republic. Diabetes Res Clin Pract. 2021;172:108621. doi:10.1016/j.diabres.2020.108621 | Focus on diabetic foot ulcer recurrence. No data on the prevalence or incidence of diabetic foot disease were available |
| Paisey, 2019 | Paisey RB, Paisey CF, Abbott A, Walker D. Diabetic foot ulcer incidence and outcome over 18 years: reduction in new ulcers and improved survival in younger patients without peripheral vascular disease. Diabet Med. 2019;29(Suppl 1). doi:10.1111/dme.29_13883 | Conference abstract |
| Prompers, 2007 | Prompers L, Huijberts M, Apelqvist J, et al. High prevalence of ischaemia, infection and serious comorbidity in patients with diabetic foot disease in Europe. Baseline results from the Eurodiale study. Diabetologia. 2007;50(1):18-25. doi:10.1007/s00125-006-0491-1 | No data on the prevalence or incidence of diabetic foot disease were available |
| Putro, 2021 | Putro MFU, Putrinadia A, Verinda A, Budi M, Erawan I, Tresna IGNA. Prevalence of hypertensive crisis as a comorbid among patients with uncontrolled type 2 diabetic mellitus in emergency department, Karanganyar General Hospital: a cross-sectional study. J Hypertens. 2021;39(Suppl 1):e9. doi:10.1097/01.hjh.0000752488.92301.d9. | Conference abstract |
| Rezaei, 2018 | Rezaei Shirinabadi P, Nossoohi Dehnavi MH, Taghipour Zahir S, Hossaini S, Khalili S, Shafiei M. Prevalence of diabetic foot in 2009-2013 in a province. Virchows Arch. 2018;473(Suppl 1):S1-S340 | Conference abstract |
| Rokkjær, 2020 | Rokkjær J, Werkman W, Eijkelkamp N, van den Berg J, Vestergard P, Jensen M, Klungel O, de Vries F, Nielsen J, Driessen J. Incidence of first diabetic foot ulcer, hospitalisation and mortality in people with diabetes from the primary care setting in the United Kingdom [ePoster #828_PDF]. Presented at: EASD Annual Meeting; September 24, 2020; Virtual Meeting. Session: PS 77 Focus on diabetic foot ulcers. Available at: https://easd.org/media-centre/#. Accessed February 5, 2025. | Conference abstract |
| Russo, 2023 | Russo MP, Grande-Ratti MF, Burgos MA, Molaro AA, Bonella MB. Prevalence of diabetes, epidemiological characteristics and vascular complications. Prevalencia de diabetes, características epidemiológicas y complicaciones vasculares. Arch Cardiol Mex. 2023;93(1):30-36. doi:10.24875/ACM.21000410 | Not written in English |
| Salvotelli, 2015 | Salvotelli L, Stoico V, Perrone F, et al. Prevalence of neuropathy in type 2 diabetic patients and its association with other diabetes complications: The Verona Diabetic Foot Screening Program. J Diabetes Complications. 2015;29(8):1066-1070. doi:10.1016/j.jdiacomp.2015.06.014 | No data on the prevalence or incidence of diabetic foot disease were available |
| Shah, 2019 | Shah P, Connolly M, Knott A, Ledson T, Srinivas-Shankar U. Prevalence of risk factors and burden of diabetic foot disease amongst patients on haemodialysis. Presented at: ABCD Spring Meeting 2019; Wirral University Teaching Hospital. Available at: https://abcd.care/sites/default/files/site_uploads/Abstracts/AbstractsSpring2019/Abstract_Shah_1.pdf. Accessed February 5, 2025 | Conference abstract |
| Shestakova, 2020 | Shestakova M, Vikulova O, Zheleznyakova A, Isakov M, Dedov I. Epidemiology of diabetes in Russian Federation: what has changed over 2007-2019 yr? [ePoster #339_PDF]. Presented at: EASD Annual Meeting; September 22, 2020; Virtual Meeting. Session: PS 13 Risk factors for type 2 diabetes. Available at: https://easd.org/media-centre/#. Accessed February 5, 2025. | Conference abstract |
| Spyre, 2013 | Spyre G, Gornall R, Davis J, McCarthy R, Abbott A, Paisey R. The increasing burden of diabetic foot disease: the impact on a southwest foot service over a 10-year period of sustained lowering of amputation rates but not of foot ulcer incidence. Diabet Med. 2013;30(Suppl 1):201 | Conference abstract |
| Stancu, 2022 | Stancu B, Ilyés T, Farcas M, Coman HF, Chiș BA, Andercou OA. Diabetic Foot Complications: A Retrospective Cohort Study. Int J Environ Res Public Health. 2022;20(1):187. Published 2022 Dec 23. doi:10.3390/ijerph20010187 | No data on the prevalence or incidence of diabetic foot disease were available |
| Timar, 2015 | Timar B, Chita T, Vlad M, Albai O, Vlad A, Timar R. Prevalence and risk factors for infected diabetic foot ulcers in a diabetes clinic from Romania. Diabetes. 2015;64(Suppl 1):A603-A623. doi:10.2337/db15-2374-2461. | Conference abstract |
| van der Heijden, 2015 | van der Heijden AAW, Crezee TE, van der Zwaard BC, Elders P, Nijpels G. Incidence and predictors of ulceration and amputation in a Dutch type 2 diabetes mellitus population [Abstract #61]. Presented at: EASD Annual Meeting; 2015; Stockholm, Sweden. The EMGO Institute for Health and Care Research, VU University Medical Center. Available at: https://easd.org/media-centre/#. Accessed February 5, 2025. | Conference abstract |
| Vas, 2018 | Vas PRJ, Sharma A, Cohen TS, Patel T, Thomas S, Karalliedde J. Incidence, predictors, and clinical outcomes of new onset diabetic foot ulceration after renal transplantation [Abstract #1010]. Presented at: EASD Annual Meeting; October 2, 2018; Berlin, Germany. Session: Diabetic nephropathy: Predictions are hard to make. Available at: https://easd.org/media-centre/#. Accessed February 5, 2025. | Conference abstract |
| Viswanathan, 2002 | Viswanathan V, Jasmine JJ, Snehalatha C, Ramachandran A. Prevalence of pathogens in diabetic foot infection in South Indian type 2 diabetic patients. J Assoc Physicians India. 2002;50:1013-1016. | No data on the prevalence or incidence of diabetic foot disease were available. The study was on the prevalence of pathogens in diabetic foot ulcers |
| Wang, 2022 | Wang Z, Hazlehurst J, Subramanian A, et al. Diabetic Foot Risk Classification at the Time of Type 2 Diabetes Diagnosis and Subsequent Risk of Mortality: A Population-Based Cohort Study. Front Endocrinol (Lausanne). 2022;13:888924. Published 2022 Jul 11. doi:10.3389/fendo.2022.888924 | No data on the prevalence or incidence of diabetic foot disease were available |
| Wraight, 2006 | Wraight PR, Lawrence SM, Campbell DA, Colman PG. Retrospective data for diabetic foot complications: only the tip of the iceberg?. Intern Med J. 2006;36(3):197-199. doi:10.1111/j.1445-5994.2006.01039.x | No data on the prevalence or incidence of diabetic foot disease were available. It is only possible to estimate hospital admissions for diabetic foot complications, with no information on the population at risk |
| Yazdanpanah, 2018 | Yazdanpanah L, Shahbazian H, Nazari I, et al. Incidence and Risk Factors of Diabetic Foot Ulcer: A Population-Based Diabetic Foot Cohort (ADFC Study)-Two-Year Follow-Up Study. Int J Endocrinol. 2018;2018:7631659. Published 2018 Mar 15. doi:10.1155/2018/7631659 | The data reported have already been included in another study (Yazdanpanah, 2024), which is part of this review |
| Zhang, 2015 | Zhang R, Chen H, Zhang H, Ran J. Prevalence and risk factors for diabetic foot in overweight or obese patients with type 2 diabetes. Diabetes. 2015;64(Suppl 1):A1071. | Conference abstract |
| Zhang, 2020 | Zhang Y, Lazzarini PA, McPhail SM, van Netten JJ, Armstrong DG, Pacella RE. Global Disability Burdens of Diabetes-Related Lower-Extremity Complications in 1990 and 2016. Diabetes Care. 2020;43(5):964-974. doi:10.2337/dc19-1614 | Observational, descriptive epidemiological study based on secondary data analysis from the Global Burden of Disease (GBD) 2016, using Bayesian meta-regression modeling to estimate the prevalence and disability burden of diabetes-related lower-extremity complications worldwide |

# **Table 3**. Risk of bias of prospective cohort studies included in the systematic review.

| **Author, year** | **1** | **2** | **3** | **4** | **5** | **6** | **7** | **8** | **9** | **10** | **11** | **Overall** |
| --- | --- | --- | --- | --- | --- | --- | --- | --- | --- | --- | --- | --- |
| Nelson, 1988 | Y | Y | Y | Y | Y | Y | Y | Y | Y | Y | Y | Low |
| Moss, 1992 | Y | Y | Y | Y | Y | Y | U | Y | Y | U | Y | Low |
| Rith-Najarian, 1993 | Y | Y | Y | N | N | Y | Y | Y | Y | Y | N | Moderate |
| Kästenbauer, 2001 | Y | Y | Y | Y | Y | Y | Y | Y | Y | Y | Y | Low |
| Abbott, 2002 | Y | Y | Y | Y | Y | Y | Y | Y | Y | Y | Y | Low |
| Muller, 2002 | Y | Y | Y | Y | N | Y | Y | Y | Y | Y | N | Low |
| Boyko, 2006 | Y | Y | Y | Y | Y | Y | Y | Y | Y | Y | Y | Low |
| Monami, 2009 | Y | Y | Y | Y | Y | Y | Y | Y | U | N | Y | Low |
| Williams, 2010 | Y | Y | Y | Y | Y | Y | Y | Y | Y | Y | Y | Low |
| Crawford, 2011 | Y | Y | Y | Y | Y | Y | Y | Y | Y | Y | Y | Low |
| Oe, 2014 | Y | Y | Y | N | N | Y | Y | Y | Y | Y | N | Moderate |
| Robinson, 2015 | Y | Y | Y | Y | Y | Y | Y | Y | Y | Y | Y | Low |
| Park, 2016 | Y | Y | Y | Y | Y | Y | Y | Y | Y | Y | Y | Low |
| Yun, 2016 | Y | Y | Y | Y | Y | Y | Y | Y | Y | Y | Y | Low |
| Iwase, 2018 | Y | Y | Y | Y | Y | Y | Y | Y | Y | Y | Y | Low |
| Panagoulias, 2020 | Y | Y | Y | Y | Y | Y | Y | Y | Y | Y | Y | Low |
| Bundó, 2021 | Y | Y | Y | Y | N | Y | Y | Y | Y | Y | N | Low |
| Yazdanpanah, 2024 | Y | Y | Y | Y | Y | Y | Y | Y | Y | Y | Y | Low |

# **Table 4**. Risk of bias of retrospective cohort studies included in the systematic review.

| **Author, year** | **1** | **2** | **3** | **4** | **5** | **6** | **7** | **8** | **9** | **10** | **11** | **Overall** |
| --- | --- | --- | --- | --- | --- | --- | --- | --- | --- | --- | --- | --- |
| Most, 1983 | Y | Y | N | U | N | U | Y | Y | Y | Y | N | Moderate |
| Lindegard, 1984 | Y | Y | Y | N | N | U | Y | Y | Y | Y | N | Moderate |
| Waugh, 1988 | Y | Y | Y | N | N | U | Y | Y | Y | Y | N | Moderate |
| Ebsko, 1991 | Y | Y | Y | N | N | U | Y | Y | Y | Y | N | Moderate |
| Deerochanawong, 1992 | Y | Y | Y | Y | N | Y | N | Y | Y | Y | N | Moderate |
| Farrell, 1993 | Y | Y | Y | N | N | U | Y | Y | Y | Y | N | Moderate |
| Lee, 1993 | Y | Y | Y | Y | Y | Y | Y | Y | Y | Y | Y | Low |
| Valway, 1993 | Y | Y | Y | N | N | U | Y | Y | Y | Y | N | Moderate |
| New, 1997 | Y | Y | Y | Y | N | U | Y | Y | Y | Y | N | Moderate |
| Lavery, 1999 | Y | Y | Y | U | U | U | Y | Y | Y | Y | N | Moderate |
| Ramsey, 1999 | Y | Y | Y | N | N | Y | Y | Y | Y | Y | N | Moderate |
| Holstein, 2000 | Y | Y | Y | N | N | N | Y | Y | Y | Y | N | Moderate |
| Morgan, 2000 | Y | Y | U | N | N | Y | Y | Y | Y | Y | N | Moderate |
| Lavery, 2003 | Y | Y | Y | U | N | Y | Y | Y | Y | Y | N | Moderate |
| Schofield, 2009 | Y | Y | Y | U | N | U | Y | Y | Y | Y | N | Moderate |
| Bruun, 2013 | Y | Y | Y | Y | Y | Y | Y | Y | Y | Y | Y | Low |
| Jørgensen, 2013 | Y | Y | Y | Y | N | U | Y | Y | Y | Y | N | Moderate |
| Chang, 2015 | Y | Y | Y | N | N | U | Y | Y | Y | Y | N | Moderate |
| Tomita, 2016 | Y | Y | Y | Y | Y | Y | Y | Y | Y | Y | Y | Low |
| Anderson, 2018 | Y | Y | Y | Y | Y | Y | Y | Y | Y | Y | Y | Low |
| Rasmussen, 2017 | Y | Y | Y | U | N | Y | Y | Y | Y | Y | N | Moderate |
| Heald, 2019 | Y | Y | Y | Y | Y | Y | Y | Y | Y | Y | Y | Low |
| Paisey, 2019 | Y | Y | Y | Y | N | Y | Y | Y | Y | Y | N | Low |
| Adem, 2020 | Y | Y | Y | Y | Y | Y | Y | Y | Y | Y | Y | Low |
| Chou, 2020 | Y | Y | Y | Y | U | Y | Y | Y | Y | Y | U | Low |
| Déruaz-Luyet, 2020 | Y | Y | Y | Y | N | Y | Y | Y | Y | Y | N | Low |
| Li, 2020 | Y | Y | Y | Y | Y | Y | Y | Y | Y | Y | Y | Low |
| Uivaraseanu , 2020 | Y | Y | Y | N | N | U | Y | Y | Y | Y | N | Moderate |
| Lu, 2021 | Y | Y | Y | U | N | Y | Y | Y | Y | Y | N | Moderate |
| Rossboth, 2021 | Y | Y | Y | Y | Y | Y | Y | Y | Y | Y | Y | Low |
| Tai, 2021 | Y | Y | Y | N | N | U | Y | Y | Y | Y | N | Moderate |
| Lovell, 2022 | Y | Y | Y | Y | Y | U | Y | Y | Y | Y | N | Low |
| Negussie, 2024 | Y | Y | Y | Y | Y | Y | Y | Y | Y | Y | Y | Low |

# **Table 5**. Risk of bias of cross-sectional studies included in the systematic review.

| **Author, year** | **1** | **2** | **3** | **4** | **5** | **6** | **7** | **8** | **9** | **Overall** |
| --- | --- | --- | --- | --- | --- | --- | --- | --- | --- | --- |
| Walters, 1992 | Y | Y | Y | N | Y | Y | Y | Y | Y | Low |
| Nambuya, 1996 | Y | U | U | Y | Y | Y | N | Y | Y | Moderate |
| Gulliford, 2002 | Y | Y | Y | Y | Y | Y | Y | Y | Y | Low |
| Jbour, 2003 | Y | Y | Y | Y | Y | Y | Y | Y | Y | Low |
| Nyamu, 2003 | Y | U | Y | N | Y | Y | Y | Y | Y | Low |
| Stein, 2003 | Y | U | Y | N | Y | Y | Y | N | Y | Moderate |
| Viswanathan, 2005 | Y | Y | Y | N | Y | Y | Y | Y | Y | Low |
| Ndip, 2006 | Y | Y | Y | N | Y | Y | Y | Y | Y | Low |
| Fabian, 2007 | Y | Y | Y | N | Y | Y | Y | Y | Y | Low |
| El-Nahas, 2008 | Y | Y | Y | N | Y | Y | Y | Y | Y | Low |
| Sämann, 2008 | Y | Y | Y | Y | Y | Y | Y | Y | Y | Low |
| Sinharay, 2012 | U | U | Y | N | U | Y | Y | Y | Y | Moderate |
| Assaad-Khalil, 2014 | Y | Y | Y | Y | Y | Y | Y | Y | Y | Low |
| Chiwanga, 2015 | Y | Y | Y | Y | Y | Y | Y | Y | Y | Low |
| Dòria, 2016 | Y | Y | Y | Y | Y | Y | Y | Y | Y | Low |
| Sarinnapakorn, 2016 | Y | Y | Y | N | Y | Y | Y | Y | Y | Low |
| Khan, 2017 | Y | Y | U | Y | Y | Y | Y | Y | Y | Low |
| Mariam, 2017 | Y | Y | U | Y | Y | Y | Y | Y | Y | Low |
| O’Shea, 2017 | Y | Y | Y | N | Y | Y | Y | Y | Y | Low |
| Galstyan, 2018 | Y | Y | Y | N | Y | Y | U | Y | Y | Low |
| Tindong, 2018 | Y | Y | Y | Y | Y | Y | Y | Y | Y | Low |
| Vibha, 2018 | Y | Y | Y | Y | Y | Y | Y | Y | Y | Low |
| Yazdanpanah, 2018 | Y | Y | Y | Y | Y | Y | Y | Y | Y | Low |
| Younis, 2018 | Y | Y | Y | Y | Y | Y | Y | Y | Y | Low |
| Lin, 2019 | Y | Y | Y | N | Y | Y | Y | Y | Y | Low |
| Lee, 2020 | Y | Y | Y | N | Y | Y | Y | Y | Y | Low |
| Tola, 2020 | Y | Y | Y | Y | Y | Y | Y | Y | Y | Low |
| Abdelbagi, 2021 | Y | Y | Y | Y | Y | U | Y | Y | Y | Low |
| Galvão, 2021 | Y | U | Y | Y | Y | Y | Y | Y | Y | Low |
| Akhtar, 2022 | Y | Y | Y | Y | Y | Y | Y | Y | Y | Low |
| Bundó, 2022 | Y | Y | Y | Y | Y | Y | Y | Y | Y | Low |
| El Alami, 2022 | Y | Y | Y | Y | Y | Y | U | Y | Y | Low |
| Negash, 2022 | Y | Y | Y | Y | Y | Y | Y | Y | Y | Low |
| Ponirakis, 2022 | Y | Y | Y | N | Y | Y | Y | Y | Y | Low |
| UroojNajami, 2022 | Y | Y | Y | N | Y | Y | Y | Y | Y | Low |
| Hirpa, 2023 | Y | Y | Y | Y | Y | Y | Y | Y | Y | Low |
| Kilic, 2024 | Y | Y | Y | N | Y | Y | Y | Y | Y | Low |
| Kuguyo, 2024 | Y | Y | Y | Y | Y | Y | Y | Y | Y | Low |

# **Figure 1**. Funnel plot of the incidence of ulcerations with estimated Kendall's tau coefficient to test for asymmetry that may cause publication bias.

# **Figure 2**. Funnel plot of the incidence of ulcerations, including imputed studies following the application of the Trim and Fill method.

# **Figure 3**. Funnel plot of the incidence of ulcerations, having excluded the following two highly precise studies: Lu CW et al. (2021) and Lovell L et al. (2023).

# **Figure 4**. Funnel plot of the incidence of amputations with estimated Kendall's tau coefficient to test for asymmetry that may cause publication bias.

# **Figure 5**. Forest plot showing the estimated incidence of infections.

Studies are stratified by geographical zone and coloured according to their design, with green and magenta used for prospective and retrospective studies, respectively. The summary polygon at the bottom of the plot shows the results of a fixed or random-effects model when all studies are analyzed.

**Abbreviations**: RE = Random-effects; Q: Cochran’s Q statistic, along with degrees of freedom (df) and its *p*-value; I² = Inconsistency measure; τ² = Between-study variance.

# **Figure 6**. Funnel plot of the prevalence of active ulcerations with estimated Kendall's tau coefficient to test for asymmetry that may cause publication bias.

# **Figure 7**. Funnel plot of the prevalence of historical (past) ulcerations with estimated Kendall's tau coefficient to test for asymmetry that may cause publication bias.

# **Figure 8**. Funnel plot of the prevalence of amputations with estimated Kendall's tau coefficient to test for asymmetry that may cause publication bias.

# **Figure 9**. Forest plot showing the estimated prevalence of amputations, stratified by diabetic foot identification method (i.e., diagnostic codes vs. other codes).

Studies are coloured according to the prevalence type: medium teal for annual prevalence, bright magenta for period prevalence, and burnt orange for unknown prevalence. The summary polygon below each subgroup (filled in blue) shows the results of a fixed or random-effects model for just the studies within that group. The summary polygon at the bottom of the plot shows the results of the model when all studies are analyzed. The test for subgroup difference (Q_M_) reflects the statistical significance of the subgroup covariate when included in a meta-regression model. The number of events and the total number of subjects are reported only for studies with available information.

**Abbreviations**: RE: random-effects; Q: Cochran’s Q statistic, along with degrees of freedom (df) and its *p*-value; I²: inconsistency measure; τ²: between-study variance.

# **Figure 10**. Forest plot showing the estimated prevalence of amputations, stratified by study data source type.

Studies are coloured according to the prevalence type: medium teal for annual prevalence, bright magenta for period prevalence, and burnt orange for unknown prevalence. The summary polygon below each subgroup (filled in blue) shows the results of a fixed or random-effects model for just the studies within that group. The summary polygon at the bottom of the plot shows the results of the model when all studies are analyzed. The test for subgroup difference (Q_M_) reflects the statistical significance of the subgroup covariate when included in a meta-regression model. The number of events and the total number of subjects are reported only for studies with available information.

**Abbreviations**: RE: random-effects; Q: Cochran’s Q statistic, along with degrees of freedom (df) and its *p*-value; I²: inconsistency measure; τ²: between-study variance.

# **Figure 11**. Forest plot showing the estimated prevalence of infections.

Studies are coloured according to the prevalence type: green for annual prevalence, magenta for period prevalence, and orange for unknown prevalence. The summary polygon at the bottom of the plot shows the results of a fixed or random-effects model when all studies are analyzed.

**Abbreviations**: RE = Random-effects; Q = Cochran’s Q statistic, along with degrees of freedom (df) and its p-value; I² = Inconsistency measure; τ² = Between-study variance.
